# Assessment of immunostimulatory responses to the antimiR-22 oligonucleotide compound RES-010 in human peripheral blood mononuclear cells

**DOI:** 10.3389/fphar.2023.1125654

**Published:** 2023-03-23

**Authors:** Riccardo Panella, Floriana Zanderigo, Francesca Morandini, Denise Federico, Elena Vicentini, Filippo Andreetta, Alessandro Toniolo, Sakari Kauppinen

**Affiliations:** ^1^ Center for RNA Medicine, Department of Clinical Medicine, Aalborg University, Copenhagen, Denmark; ^2^ Resalis Therapeutics S.r.l., Torino, Italy; ^3^ Aptuit (Verona) S.r.l., an Evotec Company, Campus Levi-Montalcini, Verona, Italy

**Keywords:** miRNA, inhibition, drug safetey, primary cell, toxicology, RNA Medicine, Drug development, pre-clinical development

## Abstract

microRNA-22 (miR-22) is a key regulator of lipid and energy homeostasis and represents a promising therapeutic target for NAFLD and obesity. We have previously identified a locked nucleic acid (LNA)-modified antisense oligonucleotide compound complementary to miR-22, designated as RES-010 that mediated robust inhibition of miR-22 function in cultured cells and *in vivo*. In this study we investigated the immune potential of RES-010 in human peripheral blood mononuclear cells (PBMCs). We treated fresh human peripheral blood mononuclear cells isolated from six healthy volunteers with different concentrations of the RES-010 compound and assessed its proinflammatory effects by quantifying IL-1β, IL-6, IFN-γ, TNF-α, IFN-α2a, IFN-β, IL-10, and IL-17A in the supernatants collected 24 h of treatment with RES-010. The T-cell activation markers, CD69, HLA-DR, and CD25 were evaluated by flow cytometry after 24 and 144 h of treatment, respectively, whereas cell viability was assessed after 24 h of treatment with RES-010. Our results show that RES-010 compound does not induce any significant immunostimulatory responses in human PBMCs *in vitro* compared to controls, implying that the proinflammatory potential of RES-010 is low.

## Introduction

Almost 40% of adult Americans and Europeans (Craig et al., 2019) were described as overweight in 2018 and about 13% of the entire world’s adult population was considered obese, numbers which have nearly tripled since 1975 (Database, 2022). Three of the top ten causes of death (ischemic heart disease, stroke, and diabetes mellitus) are directly correlated with obesity, which is therefore defined by WHO as the primary preventable cause of death and is considered the fifth leading cause of death in western countries. Particularly worrying is the epidemic of childhood obesity (Rice and Steiner, 2016); in 2016, approximately 42 million children under the age of five were considered overweight and over 340 million children and adolescents under the age of 20 were overweight or obese (Database, 2022). Additionally, morbid and often fatal obesity is a central manifestation of genetic disorders such as the Prader-Willi Syndrome (Butler, 2011). Fat accumulation depends on the balance between anabolic processes (such as adipogenesis) (Esteve Rafols, 2014) and catabolic processes (such as thermogenesis) (Fenzl and Kiefer, 2014).

MicroRNAs (miRNAs) are short endogenous non-coding RNAs, about ∼22 nucleotides in length, that function as post-transcriptional regulators of gene expression by repressing protein translation and promoting mRNA cleavage (Bartel, 2009). MiR-22 has been identified as a key regulator of lipid and metabolic homeostasis (Kaur et al., 2011; Wang et al., 2020), which functions by orchestrating multiple gene regulatory networks including lipid biosynthesis (Koufaris et al., 2016; Yang et al., 2021; Lopez-Riera et al., 2017; Yang et al., 2012; Zou et al., 2019), mitochondria biogenesis and brown adipose tissue differentiation (Fenzl and Kiefer, 2014; Lou et al., 2021). Furthermore, the levels of miR-22 are increased during human adipocyte differentiation and in white adipose tissue (WAT) from obese human subjects compared to WAT obtain from lean subjects. Taken together, these data highlight miR-22 as a potential therapeutic target for the treatment of obesity, fatty liver disease (NAFLD) and non-alcoholic steatohepatitis (NASH) (Thibonnier et al., 2020; Lima et al., 2021; Hu et al., 2020).

Pharmacologic inhibition of miRNAs can be achieved using single-stranded, chemically modified antisense oligonucleotides (ASOs) (Exiqon et al., 2013; Quemener et al., 2022) designated as antimiRs. The use of antimiRs to manipulate miRNA levels has shown promise in the treatment of a wide variety of human diseases ranging from inflammation and viral infections to cancer and metabolism (Esau et al., 2006; Straarup et al., 2010; Obad et al., 2011; Stenvang et al., 2012; Beck et al., 2021) We have previously screened a library of LNA-modified (Funder et al., 2020; Roberts et al., 2020; Romero-Palomo et al., 2021) antimiR oligonucleotide compounds targeting miR-22 and identified compound named RES-010, which mediated potent, dose-dependent inhibition of miR-22 in cell culture and *in vivo*. In this study we assessed the immune potential of RES-010 in human peripheral blood mononuclear cells (PBMCs) isolated from healthy volunteers as part of a series of preclinical studies aimed to assess the safety and tolerability (Minnaert et al., 2021; Walsh et al., 2020) of RES-010 as potential miR-22 targeted drug for the treatment of obesity and metabolic disorders.

## Results and discussion

**FIGURE 1:**
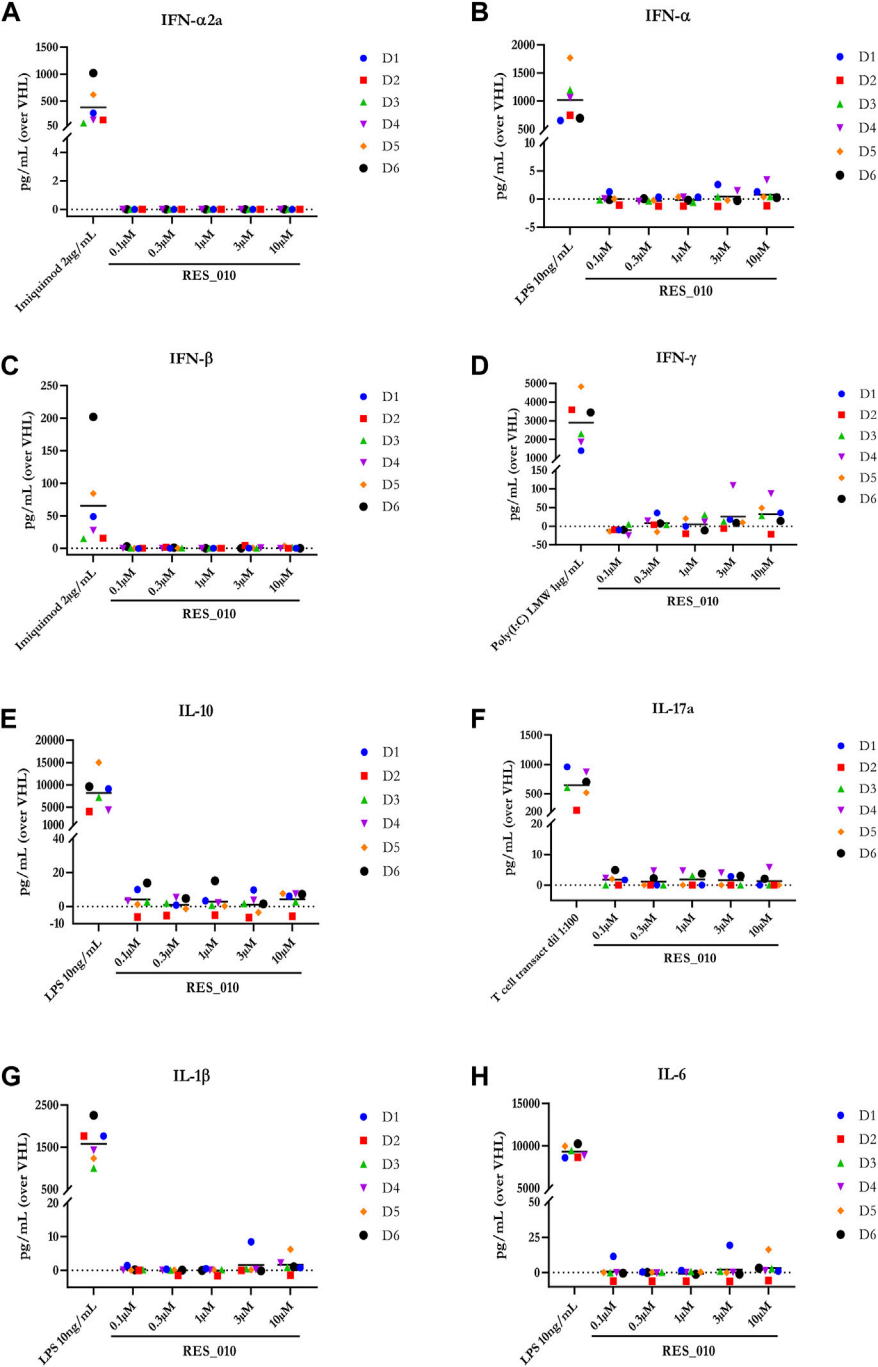
Cytokine levels after treatment of human PBMCs with RES-010. Data from individual donors are represented as median of technical triplicates, while the solid line represents the median of the entire group. Each graph represents data for a single cytokine after stimulus with positive control or with RES-010 at different concentrations. The analyzed cytokiens are; **(A)** IFN α-2a, **(B)** IFN-α, **(C)** IFN-β, **(D)** IFN-γ, **(E)** IL-10, **(F)** IL-17α, **(G)** IL1-β, and **(H)** IL-6.

MiR-22 functions as an essential regulator of lipid and metabolic homeostasis and represents a promising target for miRNA-based therapy for obesity, NAFLD and NASH. We have previously identified a potent LNA-modified antimiR compound, RES-10, for pharmacologic inhibition of miR-22 function. To assess the immune potential of RES-010 in human PBMCs, we first quantified eight cytokines (IL-1β, IL-6, IFN-γ, TNF-α, IFN-α2a, IFN-β, IL-10 and IL-17A) in the supernatants of PBMCs collected from six donors after 24 h of treatment with RES-010. Results obtained in PBMCs from all donors after treatment with positive control and with RES-010, normalized with respect to the corresponding vehicle, are reported in Tables 1–8 and are represented in Figures 1A–H whereas the concentration data for each analyte are listed in Supplementary Table S1. All the positive controls tested were efficient in inducing IFN-γ, IL-1β, IL-6, TNF-α, IL-10. Imiquimod treatment induced only IFN-α2a and IFN-β, while T-cell transact induced only IL-17A. By comparison, the levels of the assessed cytokines after treatment with RES-010 at five different concentrations were very low in all donors with most values being below level of quantification and with a slight induction was observed only for IFN-γ. None of the measured cytokines were statistically significantly different compared with the respective vehicle (Supplementary Table S2). Also, the trend analysis does not show any statistically significant differences.

**TABLE 1:** Concentration Data Vehicle Corrected (pg/mL) for Cytokine IFN-α-2a.

Treatment	Donor 1	Donor 2	Donor 3	Donor 4	Donor 5	Donor 6	Mean	SD
RES_010 0.1µM	-	-	-	-	-	-	-	-
RES_010 0.3µM	-	-	-	-	-	-	-	-
RES_010 1µM	-	-	-	-	-	-	-	-
RES_010 3µM	-	-	-	-	-	-	-	-
RES_010 10µM	-	-	-	-	-	-	-	-
LPS 10ng/mL	3.39	2.72	7.51	0.07	7.20	-	4.18	3.158
T cell transact dil 1:100	0.79	1.02	8.88	-	-	2.55	3.31	3.792
Imiquimod 2µg/mL	280.11	152.38	96.65	155.07	619.91	1020.94	387.51	363.507
Poly(I:C) LMW 1µg/mL	5.25	1.28	10.19	7.32	16.72	18.21	9.83	6.606

-: Values not quantifiable. Bold values indicates concentrations above limit of quantification (SAI1 ALQ = 38400.0 and SAI1 ALQ = 39000.0) Grey values indicates concentrations below limit of quantification (SAI7 BLQ = 9.4 and SAI7 BLQ = 9.5).

**TABLE 2:** Concentration Data Vehicle Corrected (pg/mL) for Cytokines: IFN-*β*.

Treatment	Donor 1	Donor 2	Donor 3	Donor 4	Donor 5	Donor 6	Mean	SD
RES_010 0.1µM	-0.36	-	-	-	-	2.89	1.27	2.297
RES_010 0.3µM	-	1.68	0.35	-	-	0.98	1.01	0.666
RES_010 1µM	-	0.04	-	0.10	-	-	0.07	0.047
RES_010 3µM	0.36	4.28	-	0.66	0.64	-	1.48	1.867
RES_010 10µM	-	-	1.35	-	3.83	-	2.59	1.753
LPS 10ng/mL	15.18	10.40	17.56	-	16.20	2.80	12.43	6.020
T cell transact dil 1:100	6.81	6.49	9.53	-	-	-	7.61	1.667
Imiquimod 2µg/mL	48.82	15.55	14.71	27.51	84.45	202.09	65.52	71.849
Poly(I:C) LMW 1µg/mL	16.39	0.89	22.19	12.43	29.18	36.92	19.67	12.732

-: Values not quantifiable. Bold values indicates concentrations above limit of quantification (SAI1 ALQ = 101000.0and SAI1 ALQ = 103000.0) Grey values indicates concentrations below limit of quantification (SAI7 BLQ = 24.7 and SAI7 BLQ = 25.1).

**TABLE 3:** Concentration Data Vehicle Corrected (pg/mL) for Cytokines: IFN-γ.

Treatment	Donor 1	Donor 2	Donor 3	Donor 4	Donor 5	Donor 6	Mean	SD
RES_010 0.1µM	-10.07	-9.40	4.94	-25.06	-13.86	-9.99	-10.57	9.624
RES_010 0.3µM	35.54	3.93	3.60	14.47	-15.35	7.44	8.27	16.619
RES_010 1µM	-0.46	-20.34	30.07	11.65	20.29	-11.13	5.01	19.175
RES_010 3µM	17.74	-6.13	12.37	109.63	10.08	8.65	25.39	42.031
RES_010 10µM	35.55	-21.83	27.27	87.65	49.01	13.92	31.93	36.458
LPS 10ng/mL	1964.09	3852.89	5360.27	13771.97	1928.21	3146.52	5003.99	4482.878
T cell transact dil 1:100	19428.74	**39739.97**	24117.58	**77468.43**	9386.91	**44901.73**	**35840.56**	24235.350
Imiquimod 2µg/mL	544.69	1378.16	12151.63	955.86	944.51	1574.60	2924.91	4534.596
Poly(I:C) LMW 1µg/mL	1392.91	3590.89	2290.16	1858.19	4831.58	3448.54	2902.04	1283.572

-: Values not quantifiable. Bold values indicates concentrations above limit of quantification (SAI1 ALQ = 28700.0 and SAI1 ALQ = 27000.0) Grey values indicates concentrations below limit of quantification (SAI7 BLQ = 7.0 and SAI7 BLQ = 6.6).

**TABLE 4:** Concentration Data Vehicle Corrected (pg/mL) for Cytokines: IL-1 β.

Treatment	Donor 1	Donor 2	Donor 3	Donor 4	Donor 5	Donor 6	Mean	SD
RES_010 0.1µM	1.44	-	0.15	0.09	0.09	0.16	0.39	0.593
RES_010 0.3µM	0.33	-1.53	0.07	-0.00	0.13	0.08	-0.15	0.685
RES_010 1µM	0.53	-1.59	0.15	0.08	0.19	-0.02	-0.11	0.749
RES_010 3µM	8.49	-	0.52	0.30	0.16	-0.13	1.87	3.709
RES_010 10µM	0.78	-1.45	0.88	2.22	6.25	1.05	1.62	2.563
LPS 10ng/mL	1763.75	1768.20	998.65	1426.68	1231.60	2255.83	1574.12	449.127
T cell transact dil 1:100	29.30	18.78	26.77	233.57	37.55	343.14	114.85	138.921
Imiquimod 2µg/mL	32.07	9.10	27.65	30.54	56.09	41.02	32.74	15.518
Poly(I:C) LMW 1µg/mL	22.00	25.69	3.57	3.59	4.77	3.26	10.48	10.430

-: Values not quantifiable. Bold values indicates concentrations above limit of quantification (SAI1 ALQ = 4680.0 and SAI1 ALQ = 4430.0) Grey values indicates concentrations below limit of quantification (SAI7 BLQ = 1.1 and SAI7 BLQ = 1.1).

**TABLE 5:** Concentration Data Vehicle Corrected (pg/mL) for Cytokines: IL-6.

Treatment	Donor 1	Donor 2	Donor 3	Donor 4	Donor 5	Donor 6	Mean	SD
RES_010 0.1µM	11.42	-6.09	-0.05	0.02	0.09	-0.47	0.82	5.723
RES_010 0.3µM	0.47	-6.11	0.27	-0.24	0.19	0.11	-0.88	2.571
RES_010 1µM	1.53	-6.15	0.74	0.06	0.36	-1.15	-0.77	2.783
RES_010 3µM	19.33	-6.31	0.92	0.17	-0.04	-1.03	2.17	8.800
RES_010 10µM	1.10	-5.48	2.86	1.40	16.32	3.14	3.22	7.147
LPS 10ng/mL	8602.26	8664.97	9437.63	8879.40	9960.88	10245.40	9298.43	695.194
T cell transact dil 1:100	288.35	683.66	756.69	1218.97	963.15	1295.03	867.64	373.349
Imiquimod 2µg/mL	820.71	754.35	1104.50	1538.03	**2687.95**	**2801.85**	1617.90	916.292
Poly(I:C) LMW 1µg/mL	711.81	372.02	493.82	42.81	98.11	90.02	301.43	269.565

-: Values not quantifiable. Bold values indicates concentrations above limit of quantification (SAI1 ALQ = 2100.0 and SAI1 ALQ = 2050.0) Grey values indicates concentrations below limit of quantification.

**TABLE 6:** Concentration Data Vehicle Corrected (pg/mL) for Cytokines: TNF α.

Treatment	Donor 1	Donor 2	Donor 3	Donor 4	Donor 5	Donor 6	Mean	SD
CRM0010-06 0.1µM	1.29	-1.08	-0.16	0.06	0.03	-0.09	0.01	0.756
CRM0010-06 0.3µM	0.34	-1.27	-0.32	-0.40	-0.21	0.07	-0.30	0.550
CRM0010-06 1µM	0.35	-1.25	-0.57	0.34	0.39	-0.20	-0.16	0.660
CRM0010-06 3µM	2.59	-1.28	0.39	1.48	-0.21	-0.25	0.45	1.385
CRM0010-06 10µM	1.29	-1.21	0.45	3.41	0.38	0.26	0.76	1.529
LPS 10ng/mL	656.38	746.15	1196.68	1061.77	1768.39	695.27	1020.77	425.608
T cell transact dil 1:100	1257.59	902.34	1168.46	**3868.30**	1062.15	1485.15	1624.00	1116.636
Imiquimod 2µg/mL	36.14	21.82	28.22	36.77	61.14	38.09	37.03	13.358
Poly(I:C) LMW 1µg/mL	47.15	21.01	22.87	5.75	12.34	8.14	19.55	15.151

-: Values not quantifiable. Bold values indicates concentrations above limit of quantification (SAI1 ALQ = 3540.0 and SAI1 ALQ = 3540.0) Grey values indicates concentrations below limit of quantification (SAI7 BLQ = 0.9 and SAI7 BLQ = 0.9).

**TABLE 7:** Concentration Data Vehicle Corrected (pg/mL) for Cytokines: IL-10.

Treatment	Donor 1	Donor 2	Donor 3	Donor 4	Donor 5	Donor 6	Mean	SD
RES_010 0.1µM	10.00	-6.10	2.74	3.33	1.30	13.88	4.19	6.997
RES_010 0.3µM	0.89	-5.26	2.02	5.36	-1.34	4.72	1.06	3.964
RES_010 1µM	3.42	-5.01	0.76	2.15	0.33	15.22	2.81	6.726
RES_010 3µM	9.73	-6.47	1.88	3.71	-3.44	1.57	1.16	5.659
RES_010 10µM	6.08	-5.75	2.84	7.56	7.66	7.22	4.27	5.229
LPS 10ng/mL	9098.83	4013.87	7223.07	4339.18	15031.34	9621.46	8221.29	4071.289
T cell transact dil 1:100	540.73	1523.62	1281.47	5168.86	4758.60	1036.89	2385.03	2027.921
Imiquimod 2µg/mL	2175.87	791.21	1137.64	1430.61	1579.66	1241.15	1392.69	468.948
Poly(I:C) LMW 1µg/mL	161.31	80.27	94.70	68.04	57.30	62.45	87.35	38.650

-: Values not quantifiable. Bold values indicates concentrations above limit of quantification (SAI5 and SAI6 ALQ = 40000.0) Grey values indicates concentrations below limit of quantification (SAI5 and SAI6 BLQ = 2.6).

**TABLE 8:** Concentration Data Vehicle Corrected (pg/mL) for Cytokines: IL-17A.

Treatment	Donor 1	Donor 2	Donor 3	Donor 4	Donor 5	Donor 6	Mean	SD
RES_010 0.1µM	1.67	-	-	2.34	2.00	4.93	2.74	1.489
RES_010 0.3µM	-	-	-	4.77	-	2.26	3.52	1.775
RES_010 1µM	-	-	3.09	4.74	-	3.71	3.84	0.833
RES_010 3µM	2.88	-	-	4.06	-	3.04	3.32	0.641
RES_010 10µM	-	-	-	5.82	-	2.09	3.95	2.642
LPS 10ng/mL	-	-	-	0.80	1.73	2.26	1.60	0.741
T cell transact dil 1:100	960.51	222.45	606.22	874.33	518.74	704.57	647.81	265.194
Imiquimod 2µg/mL	-	-	-	-0.69	-	-	-0.69	-
Poly(I:C) LMW 1µg/mL	0.21	-	2.31	-0.93	-	-	0.53	1.648

-: Values not quantifiable. Bold values indicates concentrations above limit of quantification (SAI5 and SAI6 ALQ = 0000.0) Grey values indicates concentrations below limit of quantification (SAI5 and SAI6 BLQ = 1.3).

**FIGURE 2:**
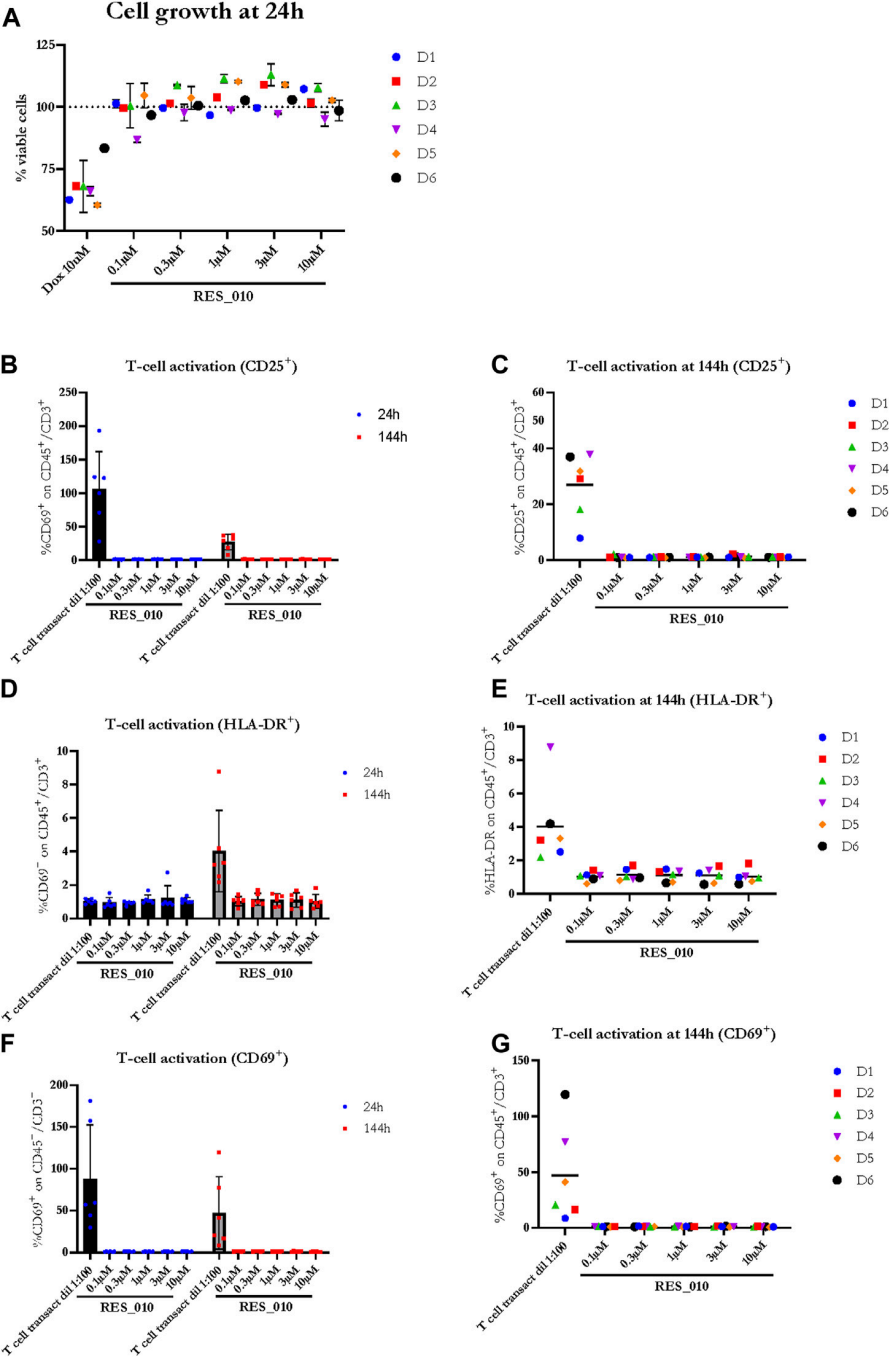
The effect of RES-010 on cell viability and T-cell activation. Cell viability for each one of the donors 24 h after RES_010 treatment is reported in **(A)**. **(B,D,F)** represent the cumulative data on % of CD69, HLA-DR and CD25 positive cells respectively on the total CD45/CD3 double positive portion at both 24 and 144 h after RES_010 treatment. **(C,E,G)** represent data at 144 h for single donors.

Next, we investigated the effect of RES-010 on cell viability 24 h after the addition of RES-010 to the media using the CellTiter-Glo^®^ assay. Results obtained after normalization are represented in Figure 2A and reported in Supplementary Table S3 with the relevant statistically significance presented in Supplementary Table S4. Reduction of cell viability after treatment with Doxorubicin positive control was evident in all donors compared to the DMSO vehicle treated samples, whereas treatment with RES-010 did not affect cell viability compared to vehicle control.

To investigate the effect of RES-010 treatment on T-cell proliferation, we analyzed two parameters for each of the T-cell activation marker, CD69, HLA-DR, CD25; 1) the geometric mean of the marker as measured by MFI, and 2) percentage of marker among the CD3^+^CD45^+^ T lymphocyte population. Each parameter was analyzed after 24 and at 144 h of treatment to better understand the kinetic on a possible pro-inflammatory activity. The results are summarized in Supplementary Table S5. The percentage of CD 25 activation is shown in Figures 2B, C while HLA-DR activation is reported in Figure 2D, E and CD69 activation is represented in Figures 2F, G. For each activation marker parameter analyzed, no dose-dependent effect of RES-010 is observed following the increase of RES-010 concentration. The trend for each donor was linear and none of the normalized values differ from the untreated controls remaining between 0.5 and 2 times the variation. The average fold change measured in six donors for each RES-010 concentration as shown by the Geometric mean for all the analyzed marked was between 0,89 and 1,20 for the 24 hour-time point and between 0,89 and 1,33 at the 144-hour time point post treatment (Figures 2B–F; Supplementary Figures S1G–H). Specifically at 24 h (Supplementary Figures S1A–F), CD69 varied between 1.02 and 1.09; HLA-DR values were between 0.96 and 1.00 and CD25 was 1.00. Treatment with RES-010 for 144 h resulted in CD69 values were between 1.03 and 1.06, HLA-DR varied between 1.01 and 1.05 and CD25 values were between 0.89 and 0.96 as show in Figures 2C, E, G. The T-cell transact treatment showed an increase of all the analyzed markers after 24 and 144 h of treatment, except for Geo Mean and % of HLA-DR, which are only weakly activated after 144 h (Figures 2B, D, F; Supplementary Figures S1J–L).

The Geo Mean for CD25, HLADR and CD69 and percentage of CD25 positive, HLADR positive and CD69 positive T Lymphocytes were subsequently selected for statistical analysis. For each parameter, a linear mixed model with Dunnett’s *post hoc* test was used to test at each timepoint the treatment effect *versus* the vehicle group. Estimated mean differences from vehicle and of *p*-values are reported in Table 9. No significant effects on T-cell proliferation were observed for RES-010.

**TABLE 9:** Statistical Result of Compound Effect vs. Vehicle.

Parameter	Timepoint (h)	Treatment	Estimate	*p*-value	Significance
Geo Mean CD69 on CD45^+^CD3^+^	24	RES_010 0.1uM	1.92	0.9999	
		RES_010 0.3uM	3.50	0.9979	
		RES_010 1uM	3.33	0.9983	
		RES_010 3uM	3.58	0.9977	
		RES_010 10uM	10.42	0.9013	
	144	RES_010 0.1uM	3.83	0.9001	
		RES_010 0.3uM	5.67	0.8009	
		RES_0101uM	6.00	0.7748	
		RES_010 3uM	8.83	0.5936	
		RES_010 10uM	5.33	0.9250	
Geo Mean HLA-DR on CD45^+^CD3^+^	24	RES_010 0.1uM	−0.25	0.9971	
		RES_010 0.3uM	−0.25	0.9969	
		RES_010 1uM	−0.33	0.9868	
		RES_010 3uM	−0.17	0.9996	
		RES_010 10uM	0.00	1.0000	
	144	RES_010 0.1uM	0.17	0.9994	
		RES_010 0.3uM	0.42	0.9723	
		RES_010 1uM	0.25	0.9971	
		RES_010 3uM	0.42	0.9769	
		RES_010 10uM	0.08	1.0000	
Geo Mean CD25 on CD45^+^CD3^+^	24	RES_010 0.1uM	0.00	1.0000	
		RES_010 0.3uM	0.00	1.0000	
		RES_010 1uM	0.00	1.0000	
		RES_010 3uM	0.00	1.0000	
		RES_010 10uM	0.00	1.0000	
	144	RES_010 0.1uM	−1.00	1.0000	
		RES_010 0.3uM	−1.75	0.9999	
		RES_010 1uM	−0.83	1.0000	
		RES_010 3uM	0.33	1.0000	
		RES_010 10uM	−2.08	0.9999	
% CD69^+^ on CD45^+^CD3^+^	24	RES_010 0.1uM	0.00	1.0000	
		RES_010 0.3uM	0.09	0.9941	
		RES_010 1uM	0.03	0.9999	
		RES_010 3uM	0.02	1.0000	
		RES_010 10uM	0.14	0.9781	
	144	RES_010 0.1uM	0.37	0.9528	
		RES_010 0.3uM	0.47	0.8654	
		RES_010 1uM	0.33	0.9555	
		RES_010 3uM	0.42	0.9507	
		RES_010 10uM	−0.00	1.0000	
% CD25^+^ on CD45^+^CD3^+^	24	RES_010 0.1uM	0.03	0.9968	
		RES_010 0.3uM	0.04	0.9949	
		RES_010 1uM	0.07	0.8512	
		RES_010 3uM	−0.03	0.9844	
		RES_010 10uM	0.03	0.9930	
	144	RES_010 0.1uM	0.38	0.9741	
		RES_010 0.3uM	0.09	0.9993	
		RES_010 1uM	0.16	0.9880	
		RES_010 3uM	0.57	0.7803	
		RES_010 10uM	0.17	0.9893	
% HLA-DR + on CD45^+^CD3^+^	24	RES_010 0.1uM	−0.10	0.8440	
		RES_010 0.3uM	−0.05	0.9915	
		RES_010 1uM	−0.01	1.0000	
		RES_010 3uM	0.06	0.9619	
		RES_010 10uM	−0.07	0.9760	
	144	RES_010 0.1uM	0.06	0.9999	
		RES_010 0.3uM	0.22	0.9777	
		RES_010 1uM	0.16	0.9930	
		RES_010 3uM	0.15	0.9958	
		RES_010 10uM	0.08	0.9998	

Significance relative to dose response linerar effect: *: *p* < 0.05; **: *p* < 0.01. Blank boxes: not statistically significant results, *p* ≥ 0.05. Significance relative to dose response linerar effect: *: *p* < 0.05; **: *p* < 0.01. Blank boxes: not statistically significant results, *p* ≥ 0.05.

Taken together, the data presented here show that RES-010 compound does not induce any significant immunostimulatory responses in human PBMCs *in vitro* compared to controls. Moreover, activation of T lymphocytes was assessed after approximately 24 and 144 h of treatment with RES-010 in the whole blood of six donors by evaluating the expression of markers CD25, CD69 and HLA DR by flow cytometry. None of the activation markers were statistically significantly increased after treatment with RES-010 indicating the immune potential of the RES-010 compound is low. The presented data are supporting further clinical development for RES-010 considering its high efficacy in modulating miR-22 levels and it reduced ability to activate any immune response.

## Materials and methods

### PBMC stimulation

Human PBMCs were isolated by Ficoll gradient centrifugation (Ficoll Plus 1.077 Cytiva Cat#17144003) starting from whole blood of six healthy donors upon blood collection in Sodium Heparin Vacutainer. In Evotec Verona Site an internal procedure regarding the blood donation from volunteer employees is applied. This procedure has been approved by an external Ethical Committee and includes a written informed consent which has to be signed for each donation. PBMCs were cultured for up to 6 days in 96-well plates at a density of 0.75 × 10^6^ cells/mL in a total volume of 200 μL, using supplemented RPMI-1640 medium (RPMI 1640 Medium, GlutaMAX™ Supplement Gibco Cat#61870-01010% FBS HI Gibco Cat#10500-064) and incubated at 37°C in a humidified, 5% CO2 atmosphere. 7.5 × 10 (Esteve Rafols, 2014) PBMCs of each donor were seeded in 96-well plate and treated with RES-010 at five different concentrations: 0.1, 0.3, 1.0, 3.0, or 10 μM, respectively, or with positive controls: LPS at 10 ng/mL (Sigma-Aldrich, Cat. N. L4399), T cell-transact diluted 1:100 (Miltenyi Biotec, Cat. N. 130-111-160), Imiquimod at 2 μg/mL (Invivogen, Cat. N. tlrl-imq) or Poly(I:C) LMW at 1 μg/mL (Invivogen, Cat. N. tlrl-picw).

In parallel to the stimuli describe above, the relative vehicle substances were also tested as negative controls, specifically NaCl 0.9% was used as vehicle control for all RES-010 treated samples, PBS (DPBS Sigma-Aldrich Cat#D8537) was used for LPS and T cell transact treatment and water was the vehicle for imiquimod and Poly(I:C) LMW samples. Additionally, Doxorubicin (Tocris, Cat. N. 2252) was tested at 10 μM to induce cell death as control for cellular viability assay. All the stimuli and vehicle substances were tested in biological duplicates.

The stimulated cells were cultured at 37°C 5% CO2 for approximately 24 and/or 144 h depending on the assay. Two independent plates were used for the two time points.

### Cytokine analysis

After approximately 24 h of stimulation, cell culture supernatants were collected and stored in aliquots at −80°C for cytokine analysis. Supernatants isolated from PBMCs stimulated with doxorubicin and its vehicle (DMSO) were not used for cytokine assays, since this stimulus was a cell death control for the CellTiter-Glo^®^ assay (Promega, Cat. N. G7572).

The cytokines IL-1β, IL-6, IFN-γ, TNF-α, IFN-α2, and IFN-β were analyzed using the MSD kit U-PLEX Biomarker Group 1 (MesoScale Discovery (MSD), Cat. N. K15067L-1). IL-10, IL-17A were analyzed using the Millipore kit Human Cytokine/Chemokine/Growth Factor Panel A Magnetic Bead Panel (EMD Millipore, Cat. N. HCYTA-60K).

Each biological duplicate, collected after PBMC stimulation, was analyzed in a single well. Raw data for IL-1β, IL-6, IFN-γ, TNF-α, IFN-α2, and IFN-β analysis were generated using MSD Discovery Workbench software platform. Concentrations for these analytes were determined from the appropriate standard curve according to the manufacturer’s instructions. Raw data for IL-10, IL-17A analysis were generated using Luminex^®^ Array Suspension System platform. Concentrations for these analytes were determined from the appropriate standard curve according to the manufacturer’s instructions by using SAS.

For each stimulus, the concentration of each analyte obtained from both replicates was normalized (by subtraction) for the mean concentration value of the corresponding vehicle. For each analyte, mean normalized value for each donor was represented using SAS. All data obtained from MSD and Luminex platform were included in the graph (even the values extrapolated by the software below the detection range).

Analytes with estimable concentrations, above the lower limit of quantification, were considered for statistical evaluation.

### Cell viability analysis

Cell viability was assessed after approximately 24 h from stimulation following the CellTiter-Glo^®^ (Promega, Cat. N. G7572) reagent instruction manual. For this analysis the cells were treated with Doxorubicin to induce cell death as positive control and with DMSO as the corresponding vehicle. Raw data (RLU) for cell viability analysis was generated by the luminometer CLARIOstar (BMGLabtech). Mean raw data obtained from medium sample was subtracted from all values obtained from the stimulated samples and corresponding vehicle. Mean vehicle values were then used for the normalization of the corresponding stimuli (stimulus/vehicle raw data%).

Mean normalized value for each donor were represented using SAS.

Raw data were used for statistical evaluation.

### T-Cell activation analysis

Activation of T lymphocytes was assessed after approximately 24 or 144 h of treatment by evaluating the expression of markers such as CD25, CD69 and HLA DR by Flow Cytometry. To this end, 10^5^ PBMCs were stained with CD45-PE-Vio770 (Miltenyi Biotec, Cat. N. 130-110-634)and CD3-APC (Miltenyi Biotec, Cat. N. 130-113-135)to select the total population of T-cells and with CD25 APC-Vio770 (Miltenyi Biotec, Cat. N. 130-123-469), CD69-PE (Miltenyi Biotec, Cat. N. 130-112-613) and HLA-DR PerCP-Vio700 (Miltenyi Biotec, Cat. N. 130-111-793) for cell activation assessment. T-cell activation was assessed using BD FACSVerse cytometer. The results and statistical parameters for each activation marker were expressed as geometric mean (MFI) for CD69, HLA-DR and CD 25 markers in the CD45^+^CD3^+^ cells as well as % of CD69HLA-DR and CD25 positive cells among CD45^+^CD3^+^ population

Each parameter was analyzed after approximately 24 and at 144 h of RES-010 treatment. Raw data derived from post-treatment of *in vitro* whole blood from six donors, as well as from all vehicles, were reported: four positive controls (Imiquimod, Poly (I:C) LMW, LPS and T cell transact) and five concentrations of compound (0.1, 0.3, 1, 3, and 10 µM). Furthermore, each raw data represented the average of the two biological replicates. All data were normalized with respect to its own vehicle by performing a ratio between treated and vehicle (fold change results).

The difference between the treatment groups and the vehicle was tested for each parameter using linear mixed model with Dunnett’s test.

### Statistical analysis

Linear mixed model with treatment as fixed effect and subject as random effect followed by Dunnnett’s test was performed to evaluate the compound differences *versus* the respective vehicle. Additional linear trend test was applied on IFNγ, IL-6 and IL-10 cytokines results to verify a dose response effect. P- values lower than 0.05 was considered statistically significant. Statistical analysis was performed using SAS software (version 9.4 SAS Institute, Cary, NC, United States).

## Data Availability

The original contributions presented in the study are included in the article/Supplementary Material, further inquiries can be directed to the corresponding author.

## Supplementary material

SUPPLEMENTARY FIGURE S1 (related to Figure 2) The effect of RES-010 on T-cell proliferation **(A‐C)** Represent the values in % of CD69, HLA-DR and CD25 positive cells from individual donors respectively on the total CD45/CD3 double positive portion 24 h after treatment with RES-010. **(D–F)** Represent the geometric mean values of CD69, HLA-DR, and CD25 positive cells respectively from individual donors on the total CD45/CD3 double positive portion at 24 h after treatment with RES_010 **(G–I)** Represent the geometric mean values of CD69, HLA-DR, and CD25 positive cells respectively from individual donors on the total CD45/CD3 double positive portion 144 h after treatment with RES_010 **(J–L)** Show the comparison between marker activation 2 and 144 h after RES-010 treatment, respectively.
