# Supplementary material for: Assessment of immunostimulatory responses to the antimiR-22 oligonucleotide compound RES-010 in human peripheral blood mononuclear cells
Source: Front Pharmacol. 2023 Mar 23;14:1125654. doi: 10.3389/fphar.2023.1125654 (PMC10076763; doi:10.3389/fphar.2023.1125654)
Supplement: Supplementary file 1 [file DataSheet2.PDF]

Supplementary Table 2 Statitical Results of Compound Effect vs Vehicle for Cytokines

			95% CI			
**Analyte**	**Treatment**	**Estimate**	**Lower Limit**	**Upper Limit**	**P-value**	**Significance**
IFNγ	RES_010 0.1µM	-10.57	-35.65	14.50	0.6386	
	RES_010 0.3µM	8.27	-29.17	45.71	0.9505	
	RES_010 1µM	5.01	-31.37	41.39	0.9931	
	RES_010 3µM	25.39	-47.32	98.11	0.7744	
	RES_010 10µM	31.93	-32.43	96.29	0.4991	
IL-6	RES_010 0.1µM	0.82	-6.90	8.54	0.9981	
	RES_010 0.3µM	-0.88	-4.04	2.27	0.8917	
	RES_010 1µM	-0.77	-4.37	2.84	0.9605	
	RES_010 3µM	2.17	-9.40	13.75	0.9761	
	RES_010 10µM	3.22	-4.93	11.37	0.7001	
IL-10	RES_010 0.1µM	4.19	-7.98	16.36	0.8043	
	RES_010 0.3µM	1.06	-6.97	9.09	0.9955	
	RES_010 1µM	2.81	-9.62	15.24	0.9549	
	RES_010 3µM	1.16	-7.86	10.19	0.9960	
	RES_010 10µM	4.27	-5.02	13.55	0.5888	

Significance relative to the treatment comparisons to the Vehicle group: *: $p<0.05$; **: $p<0.01$.
Blank boxes: not statistically significant results, $p\geq0.05$
