# Supplementary figures and images for: Assessment of immunostimulatory responses to the antimiR-22 oligonucleotide compound RES-010 in human peripheral blood mononuclear cells
Source: Front Pharmacol. 2023 Mar 23;14:1125654. doi: 10.3389/fphar.2023.1125654 (PMC10076763; doi:10.3389/fphar.2023.1125654)

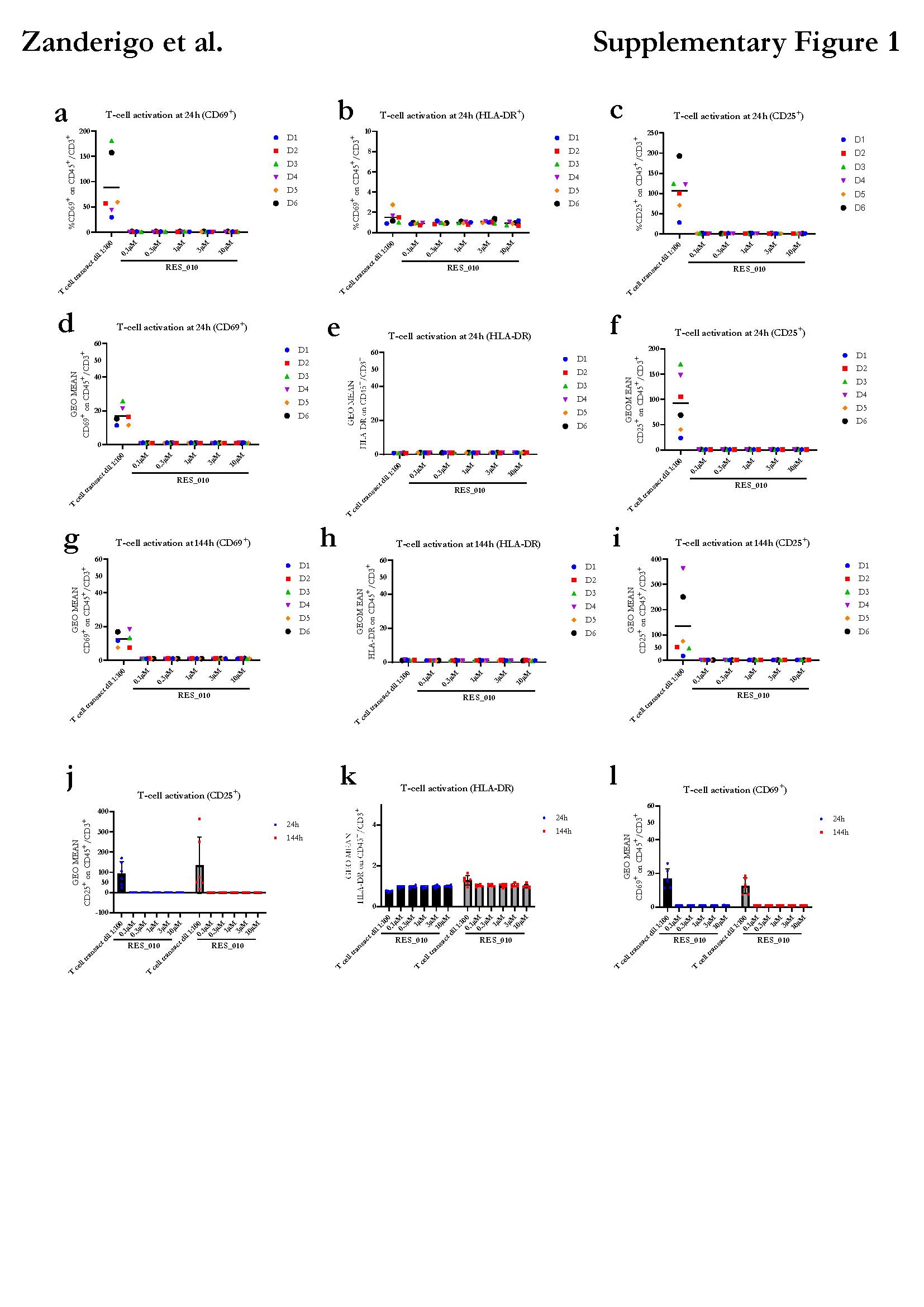

Supplement: Supplementary file 2 [file Image1.tiff]
