# Supplementary material for: Assessment of immunostimulatory responses to the antimiR-22 oligonucleotide compound RES-010 in human peripheral blood mononuclear cells
Source: Front Pharmacol. 2023 Mar 23;14:1125654. doi: 10.3389/fphar.2023.1125654 (PMC10076763; doi:10.3389/fphar.2023.1125654)
Supplement: Supplementary file 3 [file DataSheet4.pdf]

Supplementary Table 4 Cell Viability Assay Results

Control Treatment	Treatment	Donor	Rep.	RLU	Raw data corrected #	Control Raw data corrected #	% cell viability
Water	Water	1	1	22535	22467.5	22453	100.1
			2	22506	22438.5	22453	99.9
		2	1	17632	17564.5	17307.5	101.5
			2	17118	17050.5	17307.5	98.5
		3	1	18560	18492.5	18310	101.0
			2	18195	18127.5	18310	99.0
		4	1	20371	20298.5	19724	102.9
			2	19222	19149.5	19724	97.1
		5	1	17359	17286.5	17302	99.9
			2	17390	17317.5	17302	100.1
		6	1	20918	20845.5	21030	99.1
			2	21287	21214.5	21030	100.9
	Imiquimod 2µg/mL	1	1	22179	22111.5	22453	98.5
			2	22020	21952.5	22453	97.8
		2	1	18104	18036.5	17307.5	104.2
			2	18835	18767.5	17307.5	108.4
		3	1	19221	19153.5	18310	104.6
			2	19310	19242.5	18310	105.1
		4	1	20255	20182.5	19724	102.3
			2	18360	18287.5	19724	92.7
		5	1	16895	16822.5	17302	97.2
			2	16636	16563.5	17302	95.7
		6	1	21156	21083.5	21030	100.3
			2	21480	21407.5	21030	101.8
	Poly(I:C) LMW 1µg/mL	1	1	22772	22704.5	22453	101.1
			2	21739	21671.5	22453	96.5
		2	1	18439	18371.5	17307.5	106.1
			2	17843	17775.5	17307.5	102.7
		3	1	20768	20700.5	18310	113.1
			2	20007	19939.5	18310	108.9
		4	1	20570	20497.5	19724	103.9
			2	21250	21177.5	19724	107.4
		5	1	19248	19175.5	17302	110.8
			2	17226	17153.5	17302	99.1
		6	1	23175	23102.5	21030	109.9
			2	22774	22701.5	21030	107.9
PBS	PBS	1	1	21743	21675.5	21538	100.6
			2	21468	21400.5	21538	99.4
		2	1	18089	18021.5	17803	101.2
			2	17652	17584.5	17803	98.8
		3	1	19269	19201.5	19080.5	100.6
			2	19027	18959.5	19080.5	99.4
		4	1	19447	19374.5	19707.5	98.3
			2	20113	20040.5	19707.5	101.7
		5	1	17662	17589.5	17453	100.8
			2	17389	17316.5	17453	99.2
		6	1	19873	19800.5	20228	97.9
			2	20728	20655.5	20228	102.1
	LPS 10ng/mL	1	1	20274	20206.5	21538	93.8

#: Raw data have been corrected by mean of experiment medium

Supplementary Table 4 Cell Viability Assay Results

Control Treatment	Treatment	Donor	Rep.	RLU	Raw data corrected #	Control Raw data corrected #	% cell viability
PBS	LPS 10ng/mL	1	2	19403	19335.5	21538	89.8
		2	1	17519	17451.5	17803	98.0
			2	17688	17620.5	17803	99.0
		3	1	19855	19787.5	19080.5	103.7
			2	19488	19420.5	19080.5	101.8
		4	1	19826	19753.5	19707.5	100.2
			2	13246	13173.5	19707.5	66.8
		5	1	17526	17453.5	17453	100.0
			2	16675	16602.5	17453	95.1
		6	1	19881	19808.5	20228	97.9
			2	20897	20824.5	20228	102.9
	T cell transact dil 1:100	1	1	24181	24113.5	21538	112.0
			2	24407	24339.5	21538	113.0
		2	1	19730	19662.5	17803	110.4
			2	20539	20471.5	17803	115.0
		3	1	21297	21229.5	19080.5	111.3
			2	22701	22633.5	19080.5	118.6
		4	1	20299	20226.5	19707.5	102.6
			2	17408	17335.5	19707.5	88.0
		5	1	19304	19231.5	17453	110.2
			2	20241	20168.5	17453	115.6
		6	1	20953	20880.5	20228	103.2
			2	22035	21962.5	20228	108.6
basal Dox	basal Dox	1	1	28068	28000.5	27335	102.4
			2	26737	26669.5	27335	97.6
		2	1	23162	23094.5	22964	100.6
			2	22901	22833.5	22964	99.4
		3	1	23879	23811.5	26750	89.0
			2	29756	29688.5	26750	111.0
		4	1	24581	24508.5	24562	99.8
			2	24688	24615.5	24562	100.2
		5	1	21077	21004.5	21212.5	99.0
			2	21493	21420.5	21212.5	101.0
		6	1	24534	24461.5	23989.5	102.0
			2	23590	23517.5	23989.5	98.0
	Dox	1	1	17335	17267.5	27335	63.2
			2	17006	16938.5	27335	62.0
		2	1	15844	15776.5	22964	68.7
			2	15602	15534.5	22964	67.6
		3	1	16285	16217.5	26750	60.6
			2	20254	20186.5	26750	75.5
		4	1	15966	15893.5	24562	64.7
			2	16605	16532.5	24562	67.3
		5	1	12775	12702.5	21212.5	59.9
			2	13026	12953.5	21212.5	61.1
		6	1	20152	20079.5	23989.5	83.7
			2	19979	19906.5	23989.5	83.0
NaCl 0.9%	NaCl 0.9%	1	1	21325	21257.5	21195	100.3
			2	21200	21132.5	21195	99.7

#: Raw data have been corrected by mean of experiment medium

Supplementary Table 4 Cell Viability Assay Results

Control Treatment	Treatment	Donor	Rep.	RLU	Raw data corrected #	Control Raw data corrected #	% cell viability
NaCl 0.9%	NaCl 0.9%	2	1	17637	17569.5	17343.5	101.3
			2	17185	17117.5	17343.5	98.7
		3	1	18286	18218.5	18169.5	100.3
			2	18188	18120.5	18169.5	99.7
		4	1	20334	20261.5	19920	101.7
			2	19651	19578.5	19920	98.3
		5	1	17989	17916.5	16496.5	108.6
			2	15149	15076.5	16496.5	91.4
		6	1	20378	20305.5	20943	97.0
			2	21653	21580.5	20943	103.0
	RES_010 0.1µM	1	1	21296	21228.5	21195	100.2
			2	21803	21735.5	21195	102.6
		2	1	17352	17284.5	17343.5	99.7
			2	17344	17276.5	17343.5	99.6
		3	1	19471	19403.5	18169.5	106.8
			2	17177	17109.5	18169.5	94.2
		4	1	17524	17451.5	19920	87.6
			2	17215	17142.5	19920	86.1
		5	1	17908	17835.5	16496.5	108.1
			2	16756	16683.5	16496.5	101.1
		6	1	20289	20216.5	20943	96.5
			2	20359	20286.5	20943	96.9
	RES_010 0.3µM	1	1	21162	21094.5	21195	99.5
			2	21191	21123.5	21195	99.7
		2	1	17798	17730.5	17343.5	102.2
			2	17528	17460.5	17343.5	100.7
		3	1	19867	19799.5	18169.5	109.0
			2	19788	19720.5	18169.5	108.5
		4	1	20002	19929.5	19920	100.0
			2	19075	19002.5	19920	95.4
		5	1	17718	17645.5	16496.5	107.0
			2	16635	16562.5	16496.5	100.4
		6	1	21084	21011.5	20943	100.3
			2	21165	21092.5	20943	100.7
	RES_010 1µM	1	1	20641	20573.5	21195	97.1
			2	20499	20431.5	21195	96.4
		2	1	18047	17979.5	17343.5	103.7
			2	18121	18053.5	17343.5	104.1
		3	1	20087	20019.5	18169.5	110.2
			2	20524	20456.5	18169.5	112.6
		4	1	19786	19713.5	19920	99.0
			2	19718	19645.5	19920	98.6
		5	1	18303	18230.5	16496.5	110.5
			2	18208	18135.5	16496.5	109.9
		6	1	21687	21614.5	20943	103.2
			2	21460	21387.5	20943	102.1
	RES_010 3µM	1	1	21324	21256.5	21195	100.3
			2	21061	20993.5	21195	99.0
		2	1	18943	18875.5	17343.5	108.8

#: Raw data have been corrected by mean of experiment medium

Supplementary Table 4 Cell Viability Assay Results

Control Treatment	Treatment	Donor	Rep.	RLU	Raw data corrected #	Control Raw data corrected #	% cell viability
NaCl 0.9%	RES_010 3µM	2	2	18981	18913.5	17343.5	109.1
		3	1	21155	21087.5	18169.5	116.1
			2	20023	19955.5	18169.5	109.8
		4	1	19413	19340.5	19920	97.1
			2	19458	19385.5	19920	97.3
		5	1	17939	17866.5	16496.5	108.3
			2	18149	18076.5	16496.5	109.6
		6	1	21560	21487.5	20943	102.6
			2	21678	21605.5	20943	103.2
	RES_010 10µM	1	1	22877	22809.5	21195	107.6
			2	22717	22649.5	21195	106.9
		2	1	17483	17415.5	17343.5	100.4
			2	17864	17796.5	17343.5	102.6
		3	1	19442	19374.5	18169.5	106.6
			2	19855	19787.5	18169.5	108.9
		4	1	19407	19334.5	19920	97.1
			2	18605	18532.5	19920	93.0
		5	1	17114	17041.5	16496.5	103.3
			2	16916	16843.5	16496.5	102.1
		6	1	21326	21253.5	20943	101.5
			2	20094	20021.5	20943	95.6

#: Raw data have been corrected by mean of experiment medium
