# Supplementary material for: Assessment of immunostimulatory responses to the antimiR-22 oligonucleotide compound RES-010 in human peripheral blood mononuclear cells
Source: Front Pharmacol. 2023 Mar 23;14:1125654. doi: 10.3389/fphar.2023.1125654 (PMC10076763; doi:10.3389/fphar.2023.1125654)
Supplement: Supplementary file 5 [file DataSheet3.pdf]

Supplementary Table 3 Cell Viability Assay Statistical Results of Compound Effect vs Vehicle

		95% CI			
**Treatment**	**Estimate**	**Lower Limit**	**Upper Limit**	**P-value**	**Significance**
RES_010 0.1μM	-373.42	-2417.49	1670.66	0.9789	
RES_010 0.3μM	336.50	-1621.37	2294.37	0.9837	
RES_010 1μM	675.50	-1130.78	2481.78	0.7463	
RES_010 3μM	892.42	-930.82	2715.65	0.5366	
RES_010 10μM	393.75	-1766.17	2553.67	0.9791	

Significance relative to the treatment comparisons to the Vehicle group: *: $p<0.05$; **: $p<0.01$.
Blank boxes: not statistically significant results, $p\geq0.05$
