# Supplementary material for: Assessment of immunostimulatory responses to the antimiR-22 oligonucleotide compound RES-010 in human peripheral blood mononuclear cells
Source: Front Pharmacol. 2023 Mar 23;14:1125654. doi: 10.3389/fphar.2023.1125654 (PMC10076763; doi:10.3389/fphar.2023.1125654)
Supplement: Supplementary file 6 [file DataSheet1.PDF]

Supplementary Table 1 Concentration Data for Cytokines (pg/mL): IFN- α2a

Treatment	Donor	Rep. 1	Rep. 2	Mean	SD
NaCl 0.9%	D1	-	-	-	-
	D2	-	-	-	-
	D3	-	-	-	-
	D4	-	-	-	-
	D5	-	-	-	-
	D6	-	-	-	-
RES_010 0.1μM	D1	-	-	-	-
	D2	-	-	-	-
	D3	-	-	-	-
	D4	-	-	-	-
	D5	-	-	-	-
	D6	-	-	-	-
RES_010 0.3μM	D1	-	-	-	-
	D2	-	-	-	-
	D3	-	-	-	-
	D4	-	-	-	-
	D5	-	-	-	-
	D6	-	-	-	-
RES_010 1μM	D1	-	-	-	-
	D2	-	-	-	-
	D3	-	-	-	-
	D4	-	-	-	-
	D5	-	-	-	-
	D6	-	-	-	-
RES_010 3μM	D1	-	-	-	-
	D2	-	-	-	-
	D3	-	-	-	-
	D4	-	-	-	-
	D5	-	-	-	-
	D6	-	-	-	-
RES_010 10μM	D1	-	-	-	-
	D2	-	-	-	-
	D3	-	-	-	-
	D4	-	-	-	-
	D5	-	-	-	-
	D6	-	-	-	-
PBS	D1	-	-	-	-
	D2	-	-	-	-
	D3	-	-	-	-
	D4	-	-	-	-
	D5	-	-	-	-
	D6	-	-	-	-
LPS 10ng/mL	D1	6.55	0.24	3.39	4.463
	D2	3.76	1.68	2.72	1.466
	D3	11.43	3.60	7.51	5.533
	D4	0.07	-	0.07	-
	D5	7.20	-	7.20	-
	D6	-	-	-	-
T cell transact dil 1:100	D1	0.79	-	0.79	-
	D2	0.97	1.07	1.02	0.070
	D3	1.18	16.57	8.88	10.880
	D4	-	-	-	-
	D5	-	-	-	-
	D6	2.55	-	2.55	-
Water	D1	-	-	-	-
	D2	-	-	-	-
	D3	-	-	-	-
	D4	-	-	-	-
	D5	-	-	-	-
	D6	-	-	-	-
Imiquimod 2μg/mL	D1	271.94	288.29	280.11	11.558
	D2	158.01	146.76	152.38	7.956
	D3	98.05	95.24	96.65	1.981
	D4	170.90	139.24	155.07	22.386
	D5	680.82	558.99	619.91	86.145
	D6	1090.25	951.62	1020.94	98.024
Poly(I:C) LMW 1μg/mL	D1	5.97	4.53	5.25	1.023
	D2	0.92	1.63	1.28	0.502
	D3	9.59	10.78	10.19	0.841
	D4	10.23	4.40	7.32	4.126
	D5	10.79	22.64	16.72	8.380
	D6	22.98	13.45	18.21	6.738

-: Values not quantifiable.
Bold values (if any) indicates concentrations above limit of quantification. Grey values (if any) indicates concentrations below limit of quantification

Supplementary Table 1 Concentration Data for Cytokines (pg/mL): IFN- β

Treatment	Donor	Rep. 1	Rep. 2	Mean	SD
NaCl 0.9%	D1	-	2.95	2.95	-
	D2	-	-	-	-
	D3	-	-	-	-
	D4	-	0.43	0.43	-
	D5	-	-	-	-
	D6	-	-	-	-
RES_010 0.1µM	D1	-	2.59	2.59	-
	D2	-	-	-	-
	D3	-	-	-	-
	D4	-	-	-	-
	D5	-	-	-	-
	D6	-	2.89	2.89	-
RES_010 0.3µM	D1	-	-	-	-
	D2	1.68	-	1.68	-
	D3	0.35	-	0.35	-
	D4	-	-	-	-
	D5	-	-	-	-
	D6	0.32	1.64	0.98	0.930
RES_010 1µM	D1	-	-	-	-
	D2	-	0.04	0.04	-
	D3	-	-	-	-
	D4	0.54	-	0.54	-
	D5	-	-	-	-
	D6	-	-	-	-
RES_010 3µM	D1	3.31	-	3.31	-
	D2	4.28	-	4.28	-
	D3	-	-	-	-
	D4	1.15	1.05	1.10	0.071
	D5	-	0.64	0.64	-
	D6	-	-	-	-
RES_010 10µM	D1	-	-	-	-
	D2	-	-	-	-
	D3	0.74	1.96	1.35	0.859
	D4	-	-	-	-
	D5	-	3.83	3.83	-
	D6	-	-	-	-
PBS	D1	-	-	-	-
	D2	2.05	-	2.05	-
	D3	0.74	0.04	0.39	0.499
	D4	-	-	-	-
	D5	-	-	-	-
	D6	-	0.84	0.84	-
LPS 10ng/mL	D1	**25.38**	4.97	15.18	14.430
	D2	19.07	5.84	12.45	9.356
	D3	21.96	13.95	17.95	5.661
	D4	-	-	-	-
	D5	**28.48**	3.92	16.20	17.361
	D6	-	3.64	3.64	-
T cell transact dil 1:100	D1	11.40	2.23	6.81	6.481
	D2	12.72	4.36	8.54	5.908
	D3	12.80	7.03	9.92	4.078
	D4	-	-	-	-
	D5	-	-	-	-
	D6	-	-	-	-
Water	D1	-	-	-	-
	D2	-	1.78	1.78	-
	D3	0.84	1.96	1.40	0.791
	D4	-	-	-	-
	D5	-	-	-	-
	D6	0.22	-	0.22	-
Imiquimod 2µg/mL	D1	**50.39**	**47.25**	**48.82**	2.219
	D2	16.56	18.10	17.33	1.088
	D3	15.50	16.72	16.11	0.862
	D4	**26.55**	**28.48**	**27.51**	1.362
	D5	**88.82**	**80.08**	**84.45**	6.186
	D6	**206.56**	**198.05**	**202.31**	6.019
Poly(I:C) LMW 1µg/mL	D1	19.07	13.71	16.39	3.792
	D2	4.97	0.35	2.66	3.268
	D3	**25.54**	21.64	23.59	2.759
	D4	9.46	15.41	12.43	4.209
	D5	**27.47**	**30.89**	**29.18**	2.420
	D6	**34.54**	**39.74**	**37.14**	3.670

-: Values not quantifiable.
Bold values (if any) indicates concentrations above limit of quantification. Grey values (if any) indicates concentrations below limit of quantification

Supplementary Table 1 Concentration Data for Cytokines (pg/mL): IFN γ

Treatment	Donor	Rep. 1	Rep. 2	Mean	SD
NaCl 0.9%	D1	12.85	32.16	22.50	13.660
	D2	48.61	3.68	26.14	31.767
	D3	0.90	0.73	0.82	0.125
	D4	70.21	40.20	55.21	21.216
	D5	10.56	62.61	36.59	36.803
	D6	54.38	16.30	35.34	26.924
RES_010 0.1µM	D1	10.07	14.80	12.44	3.344
	D2	16.74	-	16.74	-
	D3	7.90	3.60	5.75	3.038
	D4	33.18	27.11	30.15	4.291
	D5	20.95	24.51	22.73	2.523
	D6	44.19	6.51	25.35	26.645
RES_010 0.3µM	D1	58.04	-	58.04	-
	D2	31.12	29.02	30.07	1.483
	D3	4.76	4.07	4.42	0.490
	D4	116.71	22.63	69.67	66.522
	D5	23.64	18.84	21.24	3.397
	D6	41.04	44.51	42.78	2.455
RES_010 1µM	D1	20.97	23.12	22.04	1.522
	D2	10.36	1.25	5.81	6.443
	D3	13.48	48.29	30.88	24.617
	D4	54.31	79.41	66.86	17.743
	D5	86.70	27.05	56.87	42.179
	D6	17.33	31.08	24.21	9.722
RES_010 3µM	D1	7.16	73.33	40.25	46.788
	D2	38.12	1.92	20.02	25.594
	D3	23.72	2.65	13.19	14.897
	D4	148.52	181.16	164.84	23.085
	D5	55.07	38.26	46.67	11.885
	D6	62.92	25.05	43.99	26.781
RES_010 10µM	D1	81.09	35.02	58.05	32.573
	D2	2.73	5.90	4.32	2.238
	D3	50.81	5.37	28.09	32.127
	D4	130.16	155.55	142.86	17.952
	D5	65.05	106.15	85.60	29.068
	D6	38.85	59.67	49.26	14.722
PBS	D1	12.35	54.18	33.27	29.579
	D2	75.88	22.31	49.10	37.880
	D3	3.21	6.20	4.70	2.113
	D4	91.15	58.03	74.59	23.416
	D5	60.10	30.03	45.07	21.268
	D6	40.14	7.61	23.88	23.000
LPS 10ng/mL	D1	1920.55	2074.17	1997.36	108.627
	D2	3275.24	4528.74	3901.99	886.364
	D3	6286.24	4443.72	5364.98	1302.863
	D4	18338.28	9354.83	13846.56	6352.257
	D5	2305.08	1641.47	1973.28	469.243
	D6	3336.79	3004.01	3170.40	235.310
T cell transact dil 1:100	D1	24887.70	14036.32	19462.01	7673.083
	D2	**44654.20**	**34923.94**	**39789.07**	6880.334
	D3	23064.03	25180.54	24122.29	1496.598
	D4	**83279.03**	**71807.00**	**77543.02**	8111.949
	D5	9362.79	9501.16	9431.98	97.841
	D6	**48575.54**	**41275.67**	**44925.60**	5161.788
Water	D1	69.19	28.43	48.81	28.818
	D2	51.50	28.56	40.03	16.216
	D3	8.19	5.22	6.71	2.102
	D4	33.05	9.35	21.20	16.761
	D5	12.05	80.88	46.47	48.671
	D6	17.47	8.70	13.08	6.201
Imiquimod 2µg/mL	D1	600.01	586.98	593.49	9.213
	D2	2204.78	631.59	1418.19	1112.415
	D3	444.05	23872.63	12158.34	16566.503
	D4	1195.66	758.46	977.06	309.151
	D5	1117.94	864.02	990.98	179.547
	D6	644.11	2531.25	1587.68	1334.412
Poly(I:C) LMW 1µg/mL	D1	1253.93	1629.50	1441.72	265.568
	D2	1227.56	6034.27	3630.91	3398.857
	D3	3308.06	1285.68	2296.87	1430.036
	D4	1932.88	1825.89	1879.39	75.650
	D5	6525.98	3230.10	4878.04	2330.541
	D6	3794.63	3128.62	3461.63	470.941

-: Values not quantifiable.
Bold values (if any) indicates concentrations above limit of quantification. Grey values (if any) indicates concentrations below limit of quantification

Supplementary Table 1 Concentration Data for Cytokines (pg/mL): IL-1 β

Treatment	Donor	Rep. 1	Rep. 2	Mean	SD
NaCl 0.9%	D1	0.38	0.26	0.32	0.086
	D2	1.64	-	1.64	-
	D3	0.15	0.04	0.09	0.077
	D4	0.18	0.16	0.17	0.014
	D5	0.13	0.01	0.07	0.088
	D6	0.21	0.24	0.22	0.019
RES_010 0.1µM	D1	0.54	2.99	1.77	1.733
	D2	-	-	-	-
	D3	0.19	0.29	0.24	0.074
	D4	0.30	0.21	0.26	0.059
	D5	0.12	0.20	0.16	0.056
	D6	0.57	0.20	0.38	0.261
RES_010 0.3µM	D1	-	0.66	0.66	-
	D2	0.15	0.08	0.11	0.047
	D3	0.17	0.16	0.16	0.005
	D4	0.19	0.15	0.17	0.030
	D5	0.15	0.25	0.20	0.077
	D6	0.21	0.40	0.31	0.133
RES_010 1µM	D1	0.62	1.09	0.85	0.330
	D2	0.10	0.00	0.05	0.070
	D3	0.22	0.27	0.25	0.034
	D4	0.24	0.26	0.25	0.012
	D5	0.18	0.32	0.25	0.101
	D6	-	0.20	0.20	-
RES_010 3µM	D1	2.47	15.16	8.81	8.971
	D2	-	-	-	-
	D3	1.02	0.20	0.61	0.579
	D4	0.34	0.60	0.47	0.185
	D5	0.22	0.24	0.23	0.012
	D6	0.08	0.12	0.10	0.030
RES_010 10µM	D1	1.70	0.51	1.10	0.840
	D2	0.26	0.11	0.19	0.105
	D3	1.74	0.20	0.97	1.092
	D4	0.23	4.55	2.39	3.052
	D5	12.55	0.09	6.32	8.811
	D6	0.13	2.42	1.27	1.619
PBS	D1	0.11	1.68	0.89	1.112
	D2	2.16	-	2.16	-
	D3	0.13	0.08	0.11	0.036
	D4	0.11	-	0.11	-
	D5	0.06	0.16	0.11	0.070
	D6	0.09	0.25	0.17	0.112
LPS 10ng/mL	D1	1694.11	1835.18	1764.65	99.754
	D2	1811.75	1728.96	1770.36	58.546
	D3	1076.48	921.04	998.76	109.906
	D4	1591.47	1262.11	1426.79	232.895
	D5	1348.53	1114.90	1231.72	165.200
	D6	2408.91	2103.09	2256.00	216.252
T cell transact dil 1:100	D1	29.24	31.15	30.19	1.348
	D2	24.40	17.47	20.94	4.896
	D3	38.79	14.96	26.88	16.854
	D4	228.36	239.00	233.68	7.529
	D5	43.80	31.53	37.67	8.674
	D6	381.33	305.30	343.31	53.761
Water	D1	13.73	0.11	6.92	9.624
	D2	-	-	-	-
	D3	0.05	0.16	0.11	0.076
	D4	-	-	-	-
	D5	0.19	0.17	0.18	0.014
	D6	0.12	0.18	0.15	0.044
Imiquimod 2µg/mL	D1	38.17	39.81	38.99	1.157
	D2	14.77	3.42	9.10	8.027
	D3	9.51	46.00	27.76	25.803
	D4	30.16	30.92	30.54	0.536
	D5	54.96	57.59	56.28	1.862
	D6	16.54	65.80	41.17	34.828
Poly(I:C) LMW 1µg/mL	D1	5.37	52.48	28.92	33.311
	D2	1.69	49.68	25.69	33.932
	D3	4.65	2.71	3.68	1.374
	D4	1.83	5.35	3.59	2.492
	D5	7.58	2.32	4.95	3.719
	D6	3.73	3.10	3.41	0.445

-: Values not quantifiable.
Bold values (if any) indicates concentrations above limit of quantification. Grey values (if any) indicates concentrations below limit of quantification

Supplementary Table 1 Concentration Data for Cytokines (pg/mL): IL-6

Treatment	Donor	Rep. 1	Rep. 2	Mean	SD
NaCl 0.9%	D1	4.58	4.11	4.35	0.332
	D2	12.87	0.89	6.88	8.474
	D3	1.88	1.72	1.80	0.112
	D4	1.71	1.90	1.81	0.134
	D5	2.41	2.24	2.32	0.121
	D6	0.79	2.20	1.49	0.997
RES_010 0.1µM	D1	5.07	26.48	15.77	15.140
	D2	0.82	0.76	0.79	0.044
	D3	1.64	1.87	1.75	0.162
	D4	1.92	1.74	1.83	0.124
	D5	2.57	2.26	2.41	0.219
	D6	1.11	0.93	1.02	0.126
RES_010 0.3µM	D1	4.56	5.08	4.82	0.369
	D2	0.93	0.60	0.77	0.235
	D3	1.71	2.42	2.07	0.500
	D4	1.64	1.50	1.57	0.097
	D5	2.47	2.57	2.52	0.070
	D6	0.71	2.50	1.60	1.269
RES_010 1µM	D1	5.40	6.36	5.88	0.681
	D2	0.64	0.80	0.72	0.113
	D3	1.66	3.43	2.54	1.254
	D4	2.01	1.73	1.87	0.196
	D5	2.74	2.63	2.69	0.078
	D6	0.24	0.45	0.34	0.151
RES_010 3µM	D1	8.85	38.51	23.68	20.968
	D2	0.74	0.39	0.57	0.247
	D3	3.73	1.72	2.72	1.418
	D4	1.61	2.36	1.98	0.532
	D5	2.51	2.05	2.28	0.323
	D6	0.61	0.31	0.46	0.213
RES_010 10µM	D1	6.58	4.31	5.44	1.601
	D2	1.49	1.31	1.40	0.129
	D3	8.21	1.11	4.66	5.021
	D4	2.04	4.38	3.21	1.653
	D5	35.58	1.71	18.65	23.948
	D6	0.41	8.84	4.63	5.963
PBS	D1	6.29	7.89	7.09	1.129
	D2	2.32	0.76	1.54	1.108
	D3	2.00	1.47	1.73	0.374
	D4	1.17	1.10	1.14	0.045
	D5	2.38	2.52	2.45	0.098
	D6	0.54	1.68	1.11	0.805
LPS 10ng/mL	D1	**8697.42**	**8521.28**	**8609.35**	124.552
	D2	**8974.80**	**8358.22**	**8666.51**	435.990
	D3	**9681.33**	**9197.40**	**9439.36**	342.187
	D4	**9254.06**	**8507.02**	**8880.54**	528.240
	D5	**10068.68**	**9857.99**	**9963.33**	148.980
	D6	**10336.39**	**10156.64**	**10246.52**	127.102
T cell transact dil 1:100	D1	298.27	292.61	295.44	4.003
	D2	690.08	680.33	685.20	6.891
	D3	838.61	678.24	758.43	113.399
	D4	1203.14	1237.07	1220.10	23.996
	D5	1004.31	926.89	965.60	54.748
	D6	1348.43	1243.85	1296.14	73.954
Water	D1	50.25	3.70	26.98	32.912
	D2	1.02	1.12	1.07	0.071
	D3	1.61	3.19	2.40	1.113
	D4	0.76	0.93	0.84	0.118
	D5	2.39	2.74	2.56	0.245
	D6	0.81	0.95	0.88	0.097
Imiquimod 2µg/mL	D1	791.76	903.62	847.69	79.097
	D2	767.89	742.94	755.41	17.642
	D3	913.92	1299.88	1106.90	272.919
	D4	1523.71	1554.03	1538.87	21.436
	D5	**2689.25**	**2691.79**	**2690.52**	1.797
	D6	**2690.36**	**2915.10**	**2802.73**	158.914
Poly(I:C) LMW 1µg/mL	D1	129.85	1347.71	738.78	861.161
	D2	86.24	659.93	373.08	405.658
	D3	519.45	472.99	496.22	32.856
	D4	41.93	45.36	43.65	2.427
	D5	111.36	89.99	100.67	15.110
	D6	88.72	93.07	90.89	3.075

-: Values not quantifiable.
Bold values (if any) indicates concentrations above limit of quantification. Grey values (if any) indicates concentrations below limit of quantification

Supplementary Table 1 Concentration Data for Cytokines (pg/mL): TNF α

Treatment	Donor	Rep. 1	Rep. 2	Mean	SD
NaCl 0.9%	D1	2.34	2.05	2.20	0.202
	D2	2.97	0.72	1.84	1.593
	D3	2.31	1.71	2.01	0.430
	D4	1.29	1.29	1.29	-
	D5	0.58	1.02	0.80	0.313
	D6	0.78	0.58	0.68	0.147
RES_010 0.1μM	D1	2.63	4.34	3.48	1.210
	D2	0.88	0.65	0.77	0.158
	D3	1.95	1.74	1.85	0.148
	D4	1.33	1.37	1.35	0.031
	D5	1.03	0.61	0.82	0.297
	D6	0.55	0.62	0.59	0.047
RES_010 0.3μM	D1	2.49	2.59	2.54	0.076
	D2	0.66	0.49	0.57	0.122
	D3	1.65	1.73	1.69	0.054
	D4	1.25	0.52	0.88	0.521
	D5	0.64	0.54	0.59	0.069
	D6	0.41	1.09	0.75	0.482
RES_010 1μM	D1	2.34	2.76	2.55	0.291
	D2	0.62	0.57	0.59	0.037
	D3	1.35	1.53	1.44	0.127
	D4	1.51	1.75	1.63	0.163
	D5	1.44	0.93	1.19	0.361
	D6	0.02	0.93	0.48	0.642
RES_010 3μM	D1	4.04	5.54	4.79	1.057
	D2	0.72	0.40	0.56	0.229
	D3	2.98	1.81	2.40	0.828
	D4	2.27	3.27	2.77	0.712
	D5	0.73	0.43	0.58	0.211
	D6	0.84	0.02	0.43	0.574
RES_010 10μM	D1	3.78	3.19	3.49	0.422
	D2	0.67	0.60	0.63	0.052
	D3	3.44	1.48	2.46	1.382
	D4	5.25	4.15	4.70	0.778
	D5	1.77	0.58	1.18	0.836
	D6	0.70	1.18	0.94	0.343
PBS	D1	2.50	4.26	3.38	1.246
	D2	2.64	0.66	1.65	1.400
	D3	1.94	1.75	1.84	0.130
	D4	0.78	0.52	0.65	0.184
	D5	0.95	1.09	1.02	0.093
	D6	0.37	0.17	0.27	0.141
LPS 10ng/mL	D1	676.53	642.98	659.76	23.722
	D2	778.12	717.50	747.81	42.867
	D3	1354.13	1042.93	1198.53	220.050
	D4	1266.03	858.80	1062.41	287.956
	D5	1961.91	1576.91	1769.41	272.238
	D6	731.89	659.19	695.54	51.405
T cell transact dil 1:100	D1	1290.49	1231.44	1260.96	41.751
	D2	963.60	844.39	903.99	84.298
	D3	1125.37	1215.24	1170.31	63.544
	D4	**3951.19**	**3786.70**	**3868.94**	116.315
	D5	1069.81	1056.53	1063.17	9.387
	D6	1611.32	1359.53	1485.42	178.041
Water	D1	4.59	1.79	3.19	1.979
	D2	0.80	0.74	0.77	0.048
	D3	1.70	2.23	1.97	0.376
	D4	0.15	0.23	0.19	0.055
	D5	0.89	0.69	0.79	0.146
	D6	0.61	0.32	0.46	0.202
Imiquimod 2μg/mL	D1	36.65	42.00	39.33	3.780
	D2	21.50	23.68	22.59	1.538
	D3	25.14	35.23	30.19	7.134
	D4	36.72	37.19	36.96	0.331
	D5	51.39	72.47	61.93	14.905
	D6	32.21	44.90	38.56	8.976
Poly(I:C) LMW 1μg/mL	D1	17.97	82.71	50.34	45.773
	D2	12.61	30.96	21.79	12.971
	D3	26.78	22.90	24.84	2.748
	D4	6.35	5.53	5.94	0.577
	D5	15.49	10.77	13.13	3.338
	D6	7.16	10.05	8.60	2.043

-: Values not quantifiable.
Bold values (if any) indicates concentrations above limit of quantification. Grey values (if any) indicates concentrations below limit of quantification

Supplementary Table 1 Concentration Data for Cytokines (pg/mL): IL-10

Treatment	Donor	Rep. 1	Rep. 2	Mean	SD
NaCl 0.9%	D1	9.51	9.44	9.47	0.048
	D2	10.33	-	10.33	-
	D3	4.11	3.92	4.01	0.133
	D4	4.37	11.00	7.69	4.688
	D5	10.52	7.75	9.14	1.955
	D6	18.46	11.25	14.85	5.099
RES_010 0.1µM	D1	14.84	24.11	19.47	6.561
	D2	4.74	3.73	4.23	0.711
	D3	6.95	6.55	6.75	0.280
	D4	15.42	6.61	11.02	6.235
	D5	8.90	11.97	10.43	2.175
	D6	40.21	17.26	28.74	16.227
RES_010 0.3µM	D1	14.05	6.68	10.37	5.209
	D2	6.03	4.11	5.07	1.358
	D3	7.08	4.99	6.04	1.475
	D4	16.54	9.55	13.04	4.948
	D5	8.73	6.85	7.79	1.329
	D6	18.93	20.21	19.57	0.899
RES_010 1µM	D1	11.02	14.76	12.89	2.645
	D2	4.36	6.29	5.32	1.365
	D3	4.99	4.55	4.77	0.315
	D4	5.78	13.90	9.84	5.741
	D5	9.87	9.06	9.46	0.574
	D6	23.06	37.07	30.07	9.910
RES_010 3µM	D1	13.69	24.72	19.21	7.795
	D2	-	3.86	3.86	-
	D3	-	5.90	5.90	-
	D4	9.38	13.42	11.40	2.855
	D5	6.44	4.96	5.70	1.051
	D6	9.22	23.61	16.42	10.178
RES_010 10µM	D1	14.98	16.13	15.55	0.815
	D2	4.29	4.87	4.58	0.403
	D3	9.92	3.79	6.86	4.330
	D4	19.17	11.33	15.25	5.548
	D5	27.72	5.87	16.79	15.454
	D6	19.41	24.72	22.07	3.755
PBS	D1	18.17	21.86	20.02	2.613
	D2	6.16	3.48	4.82	1.890
	D3	4.74	3.98	4.36	0.535
	D4	10.76	11.81	11.29	0.742
	D5	7.59	6.52	7.06	0.754
	D6	18.14	10.60	14.37	5.331
LPS 10ng/mL	D1	10568.95	7668.75	9118.85	2050.754
	D2	4068.09	3969.29	4018.69	69.861
	D3	7401.99	7052.87	7227.43	246.864
	D4	3837.58	4863.34	4350.46	725.324
	D5	18086.70	11990.10	15038.40	4310.942
	D6	15355.35	3916.30	9635.82	8088.631
T cell transact dil 1:100	D1	236.79	884.69	560.74	458.134
	D2	1410.19	1646.69	1528.44	167.233
	D3	1036.14	1535.52	1285.83	353.115
	D4	5030.53	5329.76	5180.14	211.586
	D5	4488.86	5042.45	4765.66	391.450
	D6	638.36	1464.16	1051.26	583.933
Water	D1	40.02	17.29	28.65	16.070
	D2	7.95	5.19	6.57	1.953
	D3	5.90	4.61	5.25	0.909
	D4	9.46	11.73	10.60	1.602
	D5	7.34	10.11	8.73	1.958
	D6	17.02	6.52	11.77	7.423
Imiquimod 2µg/mL	D1	2133.95	2275.10	2204.53	99.807
	D2	762.00	833.56	797.78	50.603
	D3	1198.57	1087.21	1142.89	78.740
	D4	1382.89	1499.51	1441.20	82.462
	D5	1595.90	1580.87	1588.39	10.624
	D6	1209.37	1296.47	1252.92	61.591
Poly(I:C) LMW 1µg/mL	D1	131.58	248.34	189.96	82.567
	D2	62.52	111.16	86.84	34.387
	D3	98.20	101.71	99.95	2.480
	D4	67.16	90.12	78.64	16.238
	D5	63.96	68.10	66.03	2.925
	D6	70.67	77.78	74.22	5.023

-: Values not quantifiable.
Bold values (if any) indicates concentrations above limit of quantification. Grey values (if any) indicates concentrations below limit of quantification

Supplementary Table 1 Concentration Data for Cytokines (pg/mL): IL-17A

Treatment	Donor	Rep. 1	Rep. 2	Mean	SD
NaCl 0.9%	D1	-	-	-	-
	D2	-	-	-	-
	D3	-	-	-	-
	D4	-	-	-	-
	D5	-	-	-	-
	D6	-	-	-	-
RES_010 0.1µM	D1	1.67	-	1.67	-
	D2	-	-	-	-
	D3	-	-	-	-
	D4	2.78	1.91	2.34	0.616
	D5	-	2.00	2.00	-
	D6	4.93	-	4.93	-
RES_010 0.3µM	D1	-	-	-	-
	D2	-	-	-	-
	D3	-	-	-	-
	D4	4.77	-	4.77	-
	D5	-	-	-	-
	D6	2.26	-	2.26	-
RES_010 1µM	D1	-	-	-	-
	D2	-	-	-	-
	D3	-	3.09	3.09	-
	D4	2.17	7.30	4.74	3.625
	D5	-	-	-	-
	D6	-	3.71	3.71	-
RES_010 3µM	D1	-	2.88	2.88	-
	D2	-	-	-	-
	D3	-	-	-	-
	D4	1.91	6.21	4.06	3.039
	D5	-	-	-	-
	D6	-	3.04	3.04	-
RES_010 10µM	D1	-	-	-	-
	D2	-	-	-	-
	D3	-	-	-	-
	D4	7.61	4.04	5.82	2.526
	D5	-	-	-	-
	D6	-	2.09	2.09	-
PBS	D1	-	-	-	-
	D2	-	-	-	-
	D3	-	-	-	-
	D4	5.17	2.44	3.80	1.936
	D5	-	-	-	-
	D6	-	-	-	-
LPS 10ng/mL	D1	-	-	-	-
	D2	-	-	-	-
	D3	-	-	-	-
	D4	5.41	3.79	4.60	1.149
	D5	1.73	-	1.73	-
	D6	-	2.26	2.26	-
T cell transact dil 1:100	D1	849.14	1071.89	960.51	157.511
	D2	237.79	207.11	222.45	21.689
	D3	603.30	609.14	606.22	4.128
	D4	856.60	899.68	878.14	30.461
	D5	592.11	445.37	518.74	103.764
	D6	779.02	630.13	704.57	105.281
Water	D1	1.96	-	1.96	-
	D2	-	-	-	-
	D3	-	-	-	-
	D4	5.41	2.61	4.01	1.984
	D5	-	-	-	-
	D6	-	-	-	-
Imiquimod 2µg/mL	D1	-	-	-	-
	D2	-	-	-	-
	D3	-	-	-	-
	D4	2.78	3.87	3.33	0.772
	D5	-	-	-	-
	D6	-	-	-	-
Poly(I:C) LMW 1µg/mL	D1	1.53	2.81	2.17	0.904
	D2	-	-	-	-
	D3	2.31	-	2.31	-
	D4	3.20	2.95	3.08	0.180
	D5	-	-	-	-
	D6	-	-	-	-

-: Values not quantifiable.
Bold values (if any) indicates concentrations above limit of quantification. Grey values (if any) indicates concentrations below limit of quantification
