# Supplementary material for: Assessment of immunostimulatory responses to the antimiR-22 oligonucleotide compound RES-010 in human peripheral blood mononuclear cells
Source: Front Pharmacol. 2023 Mar 23;14:1125654. doi: 10.3389/fphar.2023.1125654 (PMC10076763; doi:10.3389/fphar.2023.1125654)
Supplement: Supplementary file 7 [file DataSheet5.pdf]

Supplementary Table 5 Summary Results Table of the Activation Markers

Parameter	Timepoint (h)	Treatment	Control Treatment	Donor	Raw data	Control data	Fold Change	Mean Fold Change
Geo Mean CD69 on CD45+CD3+	24	Imiquimod 2µg/mL	Water	D1	264.00	100.50	2.63	2.07
				D2	200.50	107.00	1.87	2.07
				D3	135.50	96.50	1.40	2.07
				D4	218.00	124.00	1.76	2.07
				D5	352.00	150.00	2.35	2.07
				D6	300.00	125.00	2.40	2.07
		Poly(I:C) LMW 1µg/mL	Water	D1	236.50	100.50	2.35	1.94
				D2	159.50	107.00	1.49	1.94
				D3	184.00	96.50	1.91	1.94
				D4	237.00	124.00	1.91	1.94
				D5	312.50	150.00	2.08	1.94
				D6	234.50	125.00	1.88	1.94
		LPS 10ng/mL	PBS	D1	187.50	113.50	1.65	1.40
				D2	131.00	106.00	1.24	1.40
				D3	123.00	95.00	1.29	1.40
				D4	164.50	125.50	1.31	1.40
				D5	235.50	149.50	1.58	1.40
				D6	168.50	125.50	1.34	1.40
		T cell transact dil 1:100	PBS	D1	1297.50	113.50	11.43	17.04
				D2	1758.00	106.00	16.58	17.04
				D3	2456.00	95.00	25.85	17.04
				D4	2694.50	125.50	21.47	17.04
				D5	1732.50	149.50	11.59	17.04
				D6	1920.00	125.50	15.30	17.04
		RES_010 0.1uM	NaCl 0.9%	D1	110.50	95.50	1.16	1.02
				D2	108.00	108.50	1.00	1.02
				D3	96.50	97.00	0.99	1.02
				D4	125.50	128.00	0.98	1.02
				D5	150.50	151.50	0.99	1.02
				D6	127.00	126.00	1.01	1.02
		RES_010 0.3uM	NaCl 0.9%	D1	111.00	95.50	1.16	1.03
				D2	110.00	108.50	1.01	1.03
				D3	98.00	97.00	1.01	1.03
				D4	127.50	128.00	1.00	1.03
				D5	153.50	151.50	1.01	1.03
				D6	127.50	126.00	1.01	1.03
		RES_010 1uM	NaCl 0.9%	D1	110.50	95.50	1.16	1.03
				D2	110.00	108.50	1.01	1.03
				D3	99.00	97.00	1.02	1.03
				D4	127.50	128.00	1.00	1.03
				D5	153.00	151.50	1.01	1.03
				D6	126.50	126.00	1.00	1.03
		RES_010 3uM	NaCl 0.9%	D1	108.50	95.50	1.14	1.03
				D2	110.50	108.50	1.02	1.03
				D3	99.00	97.00	1.02	1.03
				D4	128.50	128.00	1.00	1.03
				D5	153.50	151.50	1.01	1.03
				D6	128.00	126.00	1.02	1.03
		RES_010 10uM	NaCl 0.9%	D1	111.00	95.50	1.16	1.09
				D2	109.50	108.50	1.01	1.09
				D3	98.50	97.00	1.02	1.09
				D4	156.50	128.00	1.22	1.09
				D5	161.50	151.50	1.07	1.09
				D6	132.00	126.00	1.05	1.09

Supplementary Table 5 Summary Results Table of the Activation Markers

Parameter	Timepoint (h)	Treatment	Control Treatment	Donor	Raw data	Control data	Fold Change	Mean Fold Change
Geo Mean CD69 on CD45+CD3+	144	Imiquimod 2µg/mL	Water	D1	137.50	137.00	1.00	0.94
				D2	130.00	140.50	0.93	0.94
				D3	113.00	128.50	0.88	0.94
				D4	136.50	147.00	0.93	0.94
				D5	145.00	163.00	0.89	0.94
				D6	143.50	138.50	1.04	0.94
		Poly(I:C) LMW 1µg/mL	Water	D1	186.00	137.00	1.36	1.10
				D2	144.00	140.50	1.02	1.10
				D3	132.50	128.50	1.03	1.10
				D4	153.50	147.00	1.04	1.10
				D5	170.50	163.00	1.05	1.10
				D6	155.00	138.50	1.12	1.10
		LPS 10ng/mL	PBS	D1	137.50	194.00	0.71	0.86
				D2	124.50	139.50	0.89	0.86
				D3	113.00	134.00	0.84	0.86
				D4	135.00	145.00	0.93	0.86
				D5	148.00	165.00	0.90	0.86
				D6	124.50	139.50	0.89	0.86
		T cell transact dil 1:100	PBS	D1	2268.00	194.00	11.69	12.60
				D2	1061.00	139.50	7.61	12.60
				D3	1784.00	134.00	13.31	12.60
				D4	2694.50	145.00	18.58	12.60
				D5	1232.00	165.00	7.47	12.60
				D6	2363.00	139.50	16.94	12.60
		RES_010 0.1uM	NaCl 0.9%	D1	144.00	139.50	1.03	1.03
				D2	144.50	137.50	1.05	1.03
				D3	141.00	130.00	1.08	1.03
				D4	142.50	145.50	0.98	1.03
				D5	158.00	158.50	1.00	1.03
				D6	139.00	135.00	1.03	1.03
		RES_010 0.3uM	NaCl 0.9%	D1	152.00	139.50	1.09	1.04
				D2	150.50	137.50	1.09	1.04
				D3	133.00	130.00	1.02	1.04
				D4	146.00	145.50	1.00	1.04
				D5	161.00	158.50	1.02	1.04
				D6	137.50	135.00	1.02	1.04
		RES_010 1uM	NaCl 0.9%	D1	147.50	139.50	1.06	1.04
				D2	151.50	137.50	1.10	1.04
				D3	132.00	130.00	1.02	1.04
				D4	149.00	145.50	1.02	1.04
				D5	162.50	158.50	1.03	1.04
				D6	139.50	135.00	1.03	1.04
		RES_010 3uM	NaCl 0.9%	D1	147.00	139.50	1.05	1.06
				D2	165.00	137.50	1.20	1.06
				D3	130.00	130.00	1.00	1.06
				D4	148.00	145.50	1.02	1.06
				D5	164.50	158.50	1.04	1.06
				D6	144.50	135.00	1.07	1.06
		RES_010 10uM	NaCl 0.9%	D1	138.50	139.50	0.99	1.04
				D2	158.00	137.50	1.15	1.04
				D3	123.00	130.00	0.95	1.04
				D4	148.50	145.50	1.02	1.04
				D5	166.50	158.50	1.05	1.04
				D6	143.50	135.00	1.06	1.04

Supplementary Table 5 Summary Results Table of the Activation Markers

Parameter	Timepoint (h)	Treatment	Control Treatment	Donor	Raw data	Control data	Fold Change	Mean Fold Change
Geo Mean HLA-DR on CD45+CD3+	24	Imiquimod 2µg/mL	Water	D1	9.00	9.00	1.00	1.01
				D2	8.00	8.00	1.00	1.01
				D3	7.00	7.00	1.00	1.01
				D4	9.00	9.00	1.00	1.01
				D5	11.00	11.00	1.00	1.01
				D6	9.00	8.50	1.06	1.01
		Poly(I:C) LMW 1µg/mL	Water	D1	9.00	9.00	1.00	1.05
				D2	8.00	8.00	1.00	1.05
				D3	8.00	7.00	1.14	1.05
				D4	9.50	9.00	1.06	1.05
				D5	11.50	11.00	1.05	1.05
				D6	9.00	8.50	1.06	1.05
		LPS 10ng/mL	PBS	D1	9.00	9.00	1.00	1.01
				D2	8.00	8.00	1.00	1.01
				D3	7.50	7.00	1.07	1.01
				D4	9.00	9.00	1.00	1.01
				D5	11.00	11.00	1.00	1.01
				D6	9.00	9.00	1.00	1.01
		T cell transact dil 1:100	PBS	D1	6.50	9.00	0.72	0.75
				D2	6.00	8.00	0.75	0.75
				D3	5.50	7.00	0.79	0.75
				D4	7.00	9.00	0.78	0.75
				D5	8.50	11.00	0.77	0.75
				D6	6.00	9.00	0.67	0.75
		RES_010 0.1uM	NaCl 0.9%	D1	8.00	8.00	1.00	0.97
				D2	7.00	8.00	0.88	0.97
				D3	7.00	7.00	1.00	0.97
				D4	9.00	9.00	1.00	0.97
				D5	10.50	11.00	0.95	0.97
				D6	9.00	9.00	1.00	0.97
		RES_010 0.3uM	NaCl 0.9%	D1	8.50	8.00	1.06	0.97
				D2	7.00	8.00	0.88	0.97
				D3	7.00	7.00	1.00	0.97
				D4	9.00	9.00	1.00	0.97
				D5	10.50	11.00	0.95	0.97
				D6	8.50	9.00	0.94	0.97
		RES_010 1uM	NaCl 0.9%	D1	8.00	8.00	1.00	0.96
				D2	7.00	8.00	0.88	0.96
				D3	7.00	7.00	1.00	0.96
				D4	9.00	9.00	1.00	0.96
				D5	10.00	11.00	0.91	0.96
				D6	9.00	9.00	1.00	0.96
		RES_010 3uM	NaCl 0.9%	D1	8.50	8.00	1.06	0.98
				D2	7.00	8.00	0.88	0.98
				D3	7.00	7.00	1.00	0.98
				D4	9.00	9.00	1.00	0.98
				D5	10.50	11.00	0.95	0.98
				D6	9.00	9.00	1.00	0.98
		RES_010 10uM	NaCl 0.9%	D1	8.50	8.00	1.06	1.00
				D2	8.00	8.00	1.00	1.00
				D3	7.00	7.00	1.00	1.00
				D4	9.00	9.00	1.00	1.00
				D5	11.00	11.00	1.00	1.00
				D6	8.50	9.00	0.94	1.00

Supplementary Table 5 Summary Results Table of the Activation Markers

Parameter	Timepoint (h)	Treatment	Control Treatment	Donor	Raw data	Control data	Fold Change	Mean Fold Change
Geo Mean HLA-DR on CD45+CD3+	144	Imiquimod 2µg/mL	Water	D1	8.00	8.50	0.94	0.99
				D2	9.00	9.50	0.95	0.99
				D3	8.00	9.00	0.89	0.99
				D4	8.00	7.00	1.14	0.99
				D5	8.00	8.50	0.94	0.99
				D6	7.50	7.00	1.07	0.99
		Poly(I:C) LMW 1µg/mL	Water	D1	10.00	8.50	1.18	1.12
				D2	9.50	9.50	1.00	1.12
				D3	9.00	9.00	1.00	1.12
				D4	8.00	7.00	1.14	1.12
				D5	10.00	8.50	1.18	1.12
				D6	8.50	7.00	1.21	1.12
		LPS 10ng/mL	PBS	D1	8.00	10.00	0.80	0.86
				D2	8.00	9.00	0.89	0.86
				D3	7.00	9.00	0.78	0.86
				D4	7.00	7.00	1.00	0.86
				D5	7.50	9.00	0.83	0.86
				D6	6.00	7.00	0.86	0.86
		T cell transact dil 1:100	PBS	D1	12.50	10.00	1.25	1.28
				D2	12.00	9.00	1.33	1.28
				D3	12.50	9.00	1.39	1.28
				D4	11.50	7.00	1.64	1.28
				D5	8.50	9.00	0.94	1.28
				D6	8.00	7.00	1.14	1.28
		RES_010 0.1uM	NaCl 0.9%	D1	9.00	9.00	1.00	1.02
				D2	10.00	9.00	1.11	1.02
				D3	9.00	9.00	1.00	1.02
				D4	7.00	6.50	1.08	1.02
				D5	8.50	9.00	0.94	1.02
				D6	7.00	7.00	1.00	1.02
		RES_010 0.3uM	NaCl 0.9%	D1	10.00	9.00	1.11	1.05
				D2	10.00	9.00	1.11	1.05
				D3	9.00	9.00	1.00	1.05
				D4	7.00	6.50	1.08	1.05
				D5	9.00	9.00	1.00	1.05
				D6	7.00	7.00	1.00	1.05
		RES_010 1uM	NaCl 0.9%	D1	10.00	9.00	1.11	1.03
				D2	10.00	9.00	1.11	1.03
				D3	9.00	9.00	1.00	1.03
				D4	7.00	6.50	1.08	1.03
				D5	8.00	9.00	0.89	1.03
				D6	7.00	7.00	1.00	1.03
		RES_010 3uM	NaCl 0.9%	D1	10.00	9.00	1.11	1.05
				D2	10.50	9.00	1.17	1.05
				D3	8.50	9.00	0.94	1.05
				D4	7.00	6.50	1.08	1.05
				D5	9.00	9.00	1.00	1.05
				D6	7.00	7.00	1.00	1.05
		RES_010 10uM	NaCl 0.9%	D1	9.00	9.00	1.00	1.01
				D2	10.50	9.00	1.17	1.01
				D3	8.50	9.00	0.94	1.01
				D4	6.50	6.50	1.00	1.01
				D5	9.00	9.00	1.00	1.01
				D6	6.50	7.00	0.93	1.01

Supplementary Table 5 Summary Results Table of the Activation Markers

Parameter	Timepoint (h)	Treatment	Control Treatment	Donor	Raw data	Control data	Fold Change	Mean Fold Change
Geo Mean CD25 on CD45+CD3+	24	Imiquimod 2µg/mL	Water	D1	3.00	2.50	1.20	1.00
				D2	2.00	2.00	1.00	1.00
				D3	2.00	2.00	1.00	1.00
				D4	1.00	1.00	1.00	1.00
				D5	2.00	2.50	0.80	1.00
				D6	1.00	1.00	1.00	1.00
		Poly(I:C) LMW 1µg/mL	Water	D1	2.00	2.50	0.80	0.93
				D2	2.00	2.00	1.00	0.93
				D3	2.00	2.00	1.00	0.93
				D4	1.00	1.00	1.00	0.93
				D5	2.00	2.50	0.80	0.93
				D6	1.00	1.00	1.00	0.93
		LPS 10ng/mL	PBS	D1	3.00	3.00	1.00	0.97
				D2	2.00	2.00	1.00	0.97
				D3	2.00	2.00	1.00	0.97
				D4	1.00	1.00	1.00	0.97
				D5	2.00	2.50	0.80	0.97
				D6	1.00	1.00	1.00	0.97
		T cell transact dil 1:100	PBS	D1	71.50	3.00	23.83	92.74
				D2	210.50	2.00	105.25	92.74
				D3	339.50	2.00	169.75	92.74
				D4	148.00	1.00	148.00	92.74
				D5	101.50	2.50	40.60	92.74
				D6	69.00	1.00	69.00	92.74
		RES_010 0.1uM	NaCl 0.9%	D1	2.00	2.00	1.00	1.00
				D2	2.00	2.00	1.00	1.00
				D3	2.00	2.00	1.00	1.00
				D4	1.00	1.00	1.00	1.00
				D5	2.00	2.00	1.00	1.00
				D6	1.00	1.00	1.00	1.00
		RES_010 0.3uM	NaCl 0.9%	D1	2.00	2.00	1.00	1.00
				D2	2.00	2.00	1.00	1.00
				D3	2.00	2.00	1.00	1.00
				D4	1.00	1.00	1.00	1.00
				D5	2.00	2.00	1.00	1.00
				D6	1.00	1.00	1.00	1.00
		RES_010 1uM	NaCl 0.9%	D1	2.00	2.00	1.00	1.00
				D2	2.00	2.00	1.00	1.00
				D3	2.00	2.00	1.00	1.00
				D4	1.00	1.00	1.00	1.00
				D5	2.00	2.00	1.00	1.00
				D6	1.00	1.00	1.00	1.00
		RES_010 3uM	NaCl 0.9%	D1	2.00	2.00	1.00	1.00
				D2	2.00	2.00	1.00	1.00
				D3	2.00	2.00	1.00	1.00
				D4	1.00	1.00	1.00	1.00
				D5	2.00	2.00	1.00	1.00
				D6	1.00	1.00	1.00	1.00
		RES_01010uM	NaCl 0.9%	D1	2.00	2.00	1.00	1.00
				D2	2.00	2.00	1.00	1.00
				D3	2.00	2.00	1.00	1.00
				D4	1.00	1.00	1.00	1.00
				D5	2.00	2.00	1.00	1.00
				D6	1.00	1.00	1.00	1.00

Supplementary Table 5 Summary Results Table of the Activation Markers

Parameter	Timepoint (h)	Treatment	Control Treatment	Donor	Raw data	Control data	Fold Change	Mean Fold Change
Geo Mean CD25 on CD45+CD3+	144	Imiquimod 2µg/mL	Water	D1	32.00	52.00	0.62	0.69
				D2	25.00	30.50	0.82	0.69
				D3	22.00	40.00	0.55	0.69
				D4	5.50	6.00	0.92	0.69
				D5	5.50	12.00	0.46	0.69
				D6	4.00	5.00	0.80	0.69
		Poly(I:C) LMW 1µg/mL	Water	D1	42.00	52.00	0.81	0.82
				D2	37.50	30.50	1.23	0.82
				D3	25.50	40.00	0.64	0.82
				D4	5.00	6.00	0.83	0.82
				D5	7.00	12.00	0.58	0.82
				D6	4.00	5.00	0.80	0.82
		LPS 10ng/mL	PBS	D1	56.50	87.50	0.65	0.89
				D2	40.50	44.50	0.91	0.89
				D3	33.50	39.50	0.85	0.89
				D4	6.50	6.00	1.08	0.89
				D5	12.50	12.00	1.04	0.89
				D6	4.00	5.00	0.80	0.89
		T cell transact dil 1:100	PBS	D1	1544.50	87.50	17.65	134.53
				D2	2333.50	44.50	52.44	134.53
				D3	1895.50	39.50	47.99	134.53
				D4	2178.00	6.00	363.00	134.53
				D5	908.50	12.00	75.71	134.53
				D6	1252.00	5.00	250.40	134.53
		RES_010 0.1uM	NaCl 0.9%	D1	58.00	56.00	1.04	0.89
				D2	37.00	41.00	0.90	0.89
				D3	39.00	37.00	1.05	0.89
				D4	5.00	6.00	0.83	0.89
				D5	9.00	13.00	0.69	0.89
				D6	4.00	5.00	0.80	0.89
		RES_010 0.3uM	NaCl 0.9%	D1	55.00	56.00	0.98	0.89
				D2	35.00	41.00	0.85	0.89
				D3	38.50	37.00	1.04	0.89
				D4	5.50	6.00	0.92	0.89
				D5	9.50	13.00	0.73	0.89
				D6	4.00	5.00	0.80	0.89
		RES_010 1uM	NaCl 0.9%	D1	52.00	56.00	0.93	0.95
				D2	41.00	41.00	1.00	0.95
				D3	39.00	37.00	1.05	0.95
				D4	6.00	6.00	1.00	0.95
				D5	10.50	13.00	0.81	0.95
				D6	4.50	5.00	0.90	0.95
		RES_010 3uM	NaCl 0.9%	D1	52.50	56.00	0.94	0.96
				D2	48.50	41.00	1.18	0.96
				D3	40.00	37.00	1.08	0.96
				D4	6.00	6.00	1.00	0.96
				D5	8.50	13.00	0.65	0.96
				D6	4.50	5.00	0.90	0.96
		RES_010 10uM	NaCl 0.9%	D1	52.00	56.00	0.93	0.89
				D2	40.50	41.00	0.99	0.89
				D3	34.50	37.00	0.93	0.89
				D4	6.00	6.00	1.00	0.89
				D5	8.00	13.00	0.62	0.89
				D6	4.50	5.00	0.90	0.89

Supplementary Table 5 Summary Results Table of the Activation Markers

Parameter	Timepoint (h)	Treatment	Control Treatment	Donor	Raw data	Control data	Fold Change	Mean Fold Change
% CD69+ on CD45+CD3+	24	Imiquimod 2µg/mL	Water	D1	31.87	1.86	17.13	26.24
				D2	18.41	1.30	14.16	26.24
				D3	14.16	0.71	19.94	26.24
				D4	20.22	1.14	17.81	26.24
				D5	41.02	1.12	36.62	26.24
				D6	30.54	0.59	51.76	26.24
		Poly(I:C) LMW 1µg/mL	Water	D1	26.93	1.86	14.48	19.83
				D2	10.40	1.30	8.00	19.83
				D3	14.45	0.71	20.35	19.83
				D4	21.16	1.14	18.64	19.83
				D5	28.82	1.12	25.73	19.83
				D6	18.75	0.59	31.78	19.83
		LPS 10ng/mL	PBS	D1	19.05	2.18	8.76	10.56
				D2	5.46	1.33	4.12	10.56
				D3	5.68	0.46	12.34	10.56
				D4	10.62	1.85	5.74	10.56
				D5	20.16	1.34	15.10	10.56
				D6	8.67	0.50	17.33	10.56
		T cell transact dil 1:100	PBS	D1	64.66	2.18	29.73	88.16
				D2	75.68	1.33	57.12	88.16
				D3	83.28	0.46	181.03	88.16
				D4	81.94	1.85	44.29	88.16
				D5	79.31	1.34	59.40	88.16
				D6	78.71	0.50	157.41	88.16
		RES_010 0.1uM	NaCl 0.9%	D1	1.46	1.21	1.21	1.05
				D2	1.15	1.23	0.93	1.05
				D3	0.47	0.45	1.06	1.05
				D4	1.40	1.58	0.89	1.05
				D5	1.29	1.48	0.87	1.05
				D6	0.78	0.57	1.37	1.05
		RES_010 0.3uM	NaCl 0.9%	D1	1.58	1.21	1.31	1.14
				D2	1.25	1.23	1.01	1.14
				D3	0.54	0.45	1.20	1.14
				D4	1.64	1.58	1.04	1.14
				D5	1.25	1.48	0.84	1.14
				D6	0.84	0.57	1.46	1.14
		RES_010 1uM	NaCl 0.9%	D1	1.40	1.21	1.16	1.14
				D2	1.02	1.23	0.82	1.14
				D3	0.69	0.45	1.54	1.14
				D4	1.53	1.58	0.97	1.14
				D5	1.20	1.48	0.81	1.14
				D6	0.88	0.57	1.54	1.14
		RES_010 3uM	NaCl 0.9%	D1	1.39	1.21	1.15	1.07
				D2	0.95	1.23	0.77	1.07
				D3	0.48	0.45	1.08	1.07
				D4	1.70	1.58	1.08	1.07
				D5	1.26	1.48	0.85	1.07
				D6	0.86	0.57	1.50	1.07
		RES_010 10uM	NaCl 0.9%	D1	1.60	1.21	1.32	1.16
				D2	0.98	1.23	0.79	1.16
				D3	0.54	0.45	1.21	1.16
				D4	1.78	1.58	1.13	1.16
				D5	1.66	1.48	1.11	1.16
				D6	0.81	0.57	1.42	1.16

Supplementary Table 5 Summary Results Table of the Activation Markers

Parameter	Timepoint (h)	Treatment	Control Treatment	Donor	Raw data	Control data	Fold Change	Mean Fold Change
% CD69+ on CD45+CD3+	144	Imiquimod 2µg/mL	Water	D1	3.70	1.53	2.43	2.00
				D2	3.85	2.63	1.46	2.00
				D3	2.17	1.99	1.09	2.00
				D4	2.05	1.14	1.79	2.00
				D5	2.30	1.02	2.26	2.00
				D6	1.69	0.57	2.98	2.00
		Poly(I:C) LMW 1µg/mL	Water	D1	8.63	1.53	5.66	3.48
				D2	3.99	2.63	1.52	3.48
				D3	4.22	1.99	2.12	3.48
				D4	3.62	1.14	3.17	3.48
				D5	3.55	1.02	3.50	3.48
				D6	2.79	0.57	4.93	3.48
		LPS 10ng/mL	PBS	D1	1.53	7.34	0.21	0.66
				D2	1.04	2.25	0.46	0.66
				D3	0.66	2.80	0.23	0.66
				D4	1.15	0.87	1.34	0.66
				D5	0.83	0.97	0.86	0.66
				D6	0.45	0.53	0.84	0.66
		T cell transact dil 1:100	PBS	D1	62.81	7.34	8.56	47.23
				D2	37.19	2.25	16.53	47.23
				D3	57.00	2.80	20.39	47.23
				D4	66.77	0.87	77.19	47.23
				D5	39.71	0.97	41.15	47.23
				D6	63.38	0.53	119.58	47.23
		RES_010 0.1uM	NaCl 0.9%	D1	2.01	1.70	1.19	1.22
				D2	2.77	2.27	1.22	1.22
				D3	3.25	2.01	1.61	1.22
				D4	0.93	0.82	1.13	1.22
				D5	0.87	0.89	0.98	1.22
				D6	0.52	0.45	1.17	1.22
		RES_010 0.3uM	NaCl 0.9%	D1	2.82	1.70	1.66	1.33
				D2	3.10	2.27	1.37	1.33
				D3	2.15	2.01	1.07	1.33
				D4	1.23	0.82	1.52	1.33
				D5	1.19	0.89	1.34	1.33
				D6	0.45	0.45	1.01	1.33
		RES_010 1uM	NaCl 0.9%	D1	2.35	1.70	1.38	1.22
				D2	3.03	2.27	1.34	1.22
				D3	2.20	2.01	1.09	1.22
				D4	1.22	0.82	1.50	1.22
				D5	0.90	0.89	1.02	1.22
				D6	0.44	0.45	0.99	1.22
		RES_010 3uM	NaCl 0.9%	D1	1.97	1.70	1.16	1.28
				D2	4.18	2.27	1.84	1.28
				D3	1.97	2.01	0.98	1.28
				D4	1.09	0.82	1.34	1.28
				D5	0.78	0.89	0.88	1.28
				D6	0.65	0.45	1.46	1.28
		RES_010 10uM	NaCl 0.9%	D1	1.49	1.70	0.88	0.97
				D2	3.29	2.27	1.45	0.97
				D3	1.42	2.01	0.70	0.97
				D4	0.76	0.82	0.93	0.97
				D5	0.61	0.89	0.69	0.97
				D6	0.53	0.45	1.19	0.97

Supplementary Table 5 Summary Results Table of the Activation Markers

Parameter	Timepoint (h)	Treatment	Control Treatment	Donor	Raw data	Control data	Fold Change	Mean Fold Change
% CD25+ on CD45+CD3+	24	Imiquimod 2µg/mL	Water	D1	0.80	0.74	1.08	1.37
				D2	0.66	0.36	1.85	1.37
				D3	0.91	0.55	1.65	1.37
				D4	0.41	0.31	1.34	1.37
				D5	0.55	0.48	1.15	1.37
				D6	0.29	0.25	1.16	1.37
		Poly(I:C) LMW 1µg/mL	Water	D1	0.80	0.74	1.08	1.21
				D2	0.54	0.36	1.51	1.21
				D3	0.70	0.55	1.26	1.21
				D4	0.39	0.31	1.26	1.21
				D5	0.43	0.48	0.89	1.21
				D6	0.32	0.25	1.28	1.21
		LPS 10ng/mL	PBS	D1	0.92	1.01	0.91	1.56
				D2	0.69	0.39	1.79	1.56
				D3	0.95	0.36	2.63	1.56
				D4	0.31	0.27	1.15	1.56
				D5	0.36	0.41	0.89	1.56
				D6	0.24	0.12	2.00	1.56
		T cell transact dil 1:100	PBS	D1	28.33	1.01	28.04	106.39
				D2	38.52	0.39	100.05	106.39
				D3	44.64	0.36	124.00	106.39
				D4	32.49	0.27	122.58	106.39
				D5	28.68	0.41	70.80	106.39
				D6	23.14	0.12	192.83	106.39
		RES_010 0.1uM	NaCl 0.9%	D1	0.58	0.43	1.34	1.07
				D2	0.22	0.36	0.61	1.07
				D3	0.56	0.36	1.56	1.07
				D4	0.30	0.27	1.09	1.07
				D5	0.44	0.46	0.95	1.07
				D6	0.19	0.23	0.84	1.07
		RES_010 0.3uM	NaCl 0.9%	D1	0.69	0.43	1.60	1.07
				D2	0.35	0.36	0.97	1.07
				D3	0.54	0.36	1.52	1.07
				D4	0.24	0.27	0.87	1.07
				D5	0.35	0.46	0.75	1.07
				D6	0.16	0.23	0.69	1.07
		RES_010 1uM	NaCl 0.9%	D1	0.65	0.43	1.50	1.20
				D2	0.35	0.36	0.97	1.20
				D3	0.57	0.36	1.61	1.20
				D4	0.39	0.27	1.44	1.20
				D5	0.39	0.46	0.84	1.20
				D6	0.19	0.23	0.84	1.20
		RES_010 3uM	NaCl 0.9%	D1	0.48	0.43	1.12	0.94
				D2	0.25	0.36	0.69	0.94
				D3	0.25	0.36	0.70	0.94
				D4	0.28	0.27	1.04	0.94
				D5	0.39	0.46	0.85	0.94
				D6	0.29	0.23	1.27	0.94
		RES_010 10uM	NaCl 0.9%	D1	0.52	0.43	1.21	1.07
				D2	0.34	0.36	0.94	1.07
				D3	0.48	0.36	1.35	1.07
				D4	0.33	0.27	1.22	1.07
				D5	0.43	0.46	0.92	1.07
				D6	0.17	0.23	0.76	1.07

Supplementary Table 5 Summary Results Table of the Activation Markers

Parameter	Timepoint (h)	Treatment	Control Treatment	Donor	Raw data	Control data	Fold Change	Mean Fold Change
% CD25+ on CD45+CD3+	144	Imiquimod 2µg/mL	Water	D1	2.80	2.28	1.23	1.25
				D2	2.84	2.20	1.29	1.25
				D3	3.23	2.73	1.19	1.25
				D4	2.21	1.73	1.28	1.25
				D5	2.12	1.66	1.27	1.25
				D6	1.75	1.43	1.22	1.25
		Poly(I:C) LMW 1µg/mL	Water	D1	3.20	2.28	1.40	1.39
				D2	4.56	2.20	2.07	1.39
				D3	4.86	2.73	1.78	1.39
				D4	2.22	1.73	1.28	1.39
				D5	1.49	1.66	0.89	1.39
				D6	1.32	1.43	0.92	1.39
		LPS 10ng/mL	PBS	D1	2.91	7.83	0.37	0.88
				D2	2.12	2.45	0.86	0.88
				D3	2.68	3.76	0.71	0.88
				D4	1.17	1.76	0.66	0.88
				D5	2.51	1.55	1.62	0.88
				D6	1.57	1.53	1.02	0.88
		T cell transact dil 1:100	PBS	D1	61.51	7.83	7.86	26.98
				D2	71.46	2.45	29.17	26.98
				D3	68.10	3.76	18.14	26.98
				D4	66.72	1.76	37.91	26.98
				D5	49.20	1.55	31.84	26.98
				D6	56.56	1.53	36.96	26.98
		RES_010 0.1uM	NaCl 0.9%	D1	2.59	2.65	0.98	1.14
				D2	1.94	2.08	0.93	1.14
				D3	5.38	2.57	2.10	1.14
				D4	1.81	1.66	1.09	1.14
				D5	1.45	1.82	0.80	1.14
				D6	1.35	1.44	0.94	1.14
		RES_010 0.3uM	NaCl 0.9%	D1	2.50	2.65	0.94	1.04
				D2	2.48	2.08	1.20	1.04
				D3	3.03	2.57	1.18	1.04
				D4	1.67	1.66	1.00	1.04
				D5	1.61	1.82	0.88	1.04
				D6	1.44	1.44	1.00	1.04
		RES_010 1uM	NaCl 0.9%	D1	2.79	2.65	1.05	1.07
				D2	2.53	2.08	1.22	1.07
				D3	2.85	2.57	1.11	1.07
				D4	1.80	1.66	1.08	1.07
				D5	1.62	1.82	0.89	1.07
				D6	1.57	1.44	1.09	1.07
		RES_010 3uM	NaCl 0.9%	D1	2.62	2.65	0.99	1.28
				D2	4.65	2.08	2.24	1.28
				D3	3.18	2.57	1.24	1.28
				D4	2.03	1.66	1.22	1.28
				D5	1.40	1.82	0.77	1.28
				D6	1.72	1.44	1.20	1.28
		RES_010 10uM	NaCl 0.9%	D1	3.02	2.65	1.14	1.07
				D2	2.49	2.08	1.20	1.07
				D3	2.98	2.57	1.16	1.07
				D4	1.81	1.66	1.09	1.07
				D5	1.45	1.82	0.80	1.07
				D6	1.49	1.44	1.04	1.07

Supplementary Table 5 Summary Results Table of the Activation Markers

Parameter	Timepoint (h)	Treatment	Control Treatment	Donor	Raw data	Control data	Fold Change	Mean Fold Change
% HLA-DR+ on CD45+CD3+	24	Imiquimod 2µg/mL	Water	D1	0.79	0.93	0.84	0.78
				D2	0.63	0.64	0.98	0.78
				D3	0.82	0.91	0.90	0.78
				D4	0.94	1.29	0.72	0.78
				D5	0.95	1.54	0.62	0.78
				D6	0.45	0.73	0.61	0.78
		Poly(I:C) LMW 1µg/mL	Water	D1	0.93	0.93	1.00	0.97
				D2	0.54	0.64	0.84	0.97
				D3	1.10	0.91	1.22	0.97
				D4	1.11	1.29	0.86	0.97
				D5	1.08	1.54	0.70	0.97
				D6	0.89	0.73	1.21	0.97
		LPS 10ng/mL	PBS	D1	1.37	1.21	1.14	1.02
				D2	0.87	0.81	1.07	1.02
				D3	1.13	0.83	1.36	1.02
				D4	1.13	1.48	0.76	1.02
				D5	1.27	1.17	1.09	1.02
				D6	0.61	0.83	0.73	1.02
		T cell transact dil 1:100	PBS	D1	1.09	1.21	0.90	1.51
				D2	1.23	0.81	1.52	1.51
				D3	0.85	0.83	1.02	1.51
				D4	2.51	1.48	1.69	1.51
				D5	3.20	1.17	2.75	1.51
				D6	0.97	0.83	1.16	1.51
		RES_010 0.1uM	NaCl 0.9%	D1	0.80	0.95	0.85	0.89
				D2	0.54	0.73	0.74	0.89
				D3	0.96	1.00	0.96	0.89
				D4	1.08	1.13	0.95	0.89
				D5	1.03	1.17	0.88	0.89
				D6	0.82	0.84	0.98	0.89
		RES_010 0.3uM	NaCl 0.9%	D1	1.12	0.95	1.19	0.95
				D2	0.61	0.73	0.84	0.95
				D3	0.97	1.00	0.97	0.95
				D4	1.07	1.13	0.95	0.95
				D5	0.98	1.17	0.83	0.95
				D6	0.80	0.84	0.95	0.95
		RES_010 1uM	NaCl 0.9%	D1	0.96	0.95	1.02	0.98
				D2	0.60	0.73	0.82	0.98
				D3	0.94	1.00	0.94	0.98
				D4	1.21	1.13	1.07	0.98
				D5	1.11	1.17	0.94	0.98
				D6	0.93	0.84	1.11	0.98
		RES_010 3uM	NaCl 0.9%	D1	0.98	0.95	1.03	1.07
				D2	0.75	0.73	1.03	1.07
				D3	0.91	1.00	0.91	1.07
				D4	1.27	1.13	1.12	1.07
				D5	1.15	1.17	0.98	1.07
				D6	1.15	0.84	1.36	1.07
		RES_010 10uM	NaCl 0.9%	D1	1.12	0.95	1.19	0.92
				D2	0.50	0.73	0.69	0.92
				D3	0.73	1.00	0.73	0.92
				D4	1.20	1.13	1.06	0.92
				D5	1.03	1.17	0.87	0.92
				D6	0.82	0.84	0.97	0.92

Supplementary Table 5 Summary Results Table of the Activation Markers

Parameter	Timepoint (h)	Treatment	Control Treatment	Donor	Raw data	Control data	Fold Change	Mean Fold Change
% HLA-DR+ on CD45+CD3+	144	Imiquimod 2µg/mL	Water	D1	0.50	0.81	0.61	0.85
				D2	0.97	1.73	0.56	0.85
				D3	0.81	2.21	0.37	0.85
				D4	1.46	0.85	1.72	0.85
				D5	0.81	0.95	0.86	0.85
				D6	0.90	0.90	1.01	0.85
		Poly(I:C) LMW 1µg/mL	Water	D1	1.82	0.81	2.25	1.59
				D2	1.92	1.73	1.11	1.59
				D3	1.93	2.21	0.87	1.59
				D4	1.62	0.85	1.91	1.59
				D5	1.78	0.95	1.88	1.59
				D6	1.38	0.90	1.54	1.59
		LPS 10ng/mL	PBS	D1	0.47	1.82	0.26	0.53
				D2	0.60	1.58	0.38	0.53
				D3	0.40	2.32	0.17	0.53
				D4	0.85	0.70	1.22	0.53
				D5	0.58	1.36	0.43	0.53
				D6	0.60	0.82	0.74	0.53
		T cell transact dil 1:100	PBS	D1	4.57	1.82	2.51	4.03
				D2	5.07	1.58	3.21	4.03
				D3	5.07	2.32	2.19	4.03
				D4	6.10	0.70	8.77	4.03
				D5	4.50	1.36	3.32	4.03
				D6	3.41	0.82	4.18	4.03
		RES_010 0.1uM	NaCl 0.9%	D1	1.34	1.18	1.14	1.04
				D2	2.12	1.49	1.42	1.04
				D3	2.18	2.03	1.08	1.04
				D4	0.73	0.66	1.10	1.04
				D5	0.90	1.48	0.61	1.04
				D6	0.65	0.72	0.90	1.04
		RES_010 0.3uM	NaCl 0.9%	D1	1.72	1.18	1.46	1.15
				D2	2.56	1.49	1.71	1.15
				D3	2.10	2.03	1.03	1.15
				D4	0.59	0.66	0.89	1.15
				D5	1.19	1.48	0.81	1.15
				D6	0.70	0.72	0.97	1.15
		RES_010 1uM	NaCl 0.9%	D1	1.74	1.18	1.48	1.12
				D2	1.98	1.49	1.33	1.12
				D3	2.35	2.03	1.16	1.12
				D4	0.91	0.66	1.37	1.12
				D5	1.04	1.48	0.70	1.12
				D6	0.48	0.72	0.66	1.12
		RES_010 3uM	NaCl 0.9%	D1	1.46	1.18	1.24	1.10
				D2	2.49	1.49	1.67	1.10
				D3	2.23	2.03	1.10	1.10
				D4	0.93	0.66	1.41	1.10
				D5	0.94	1.48	0.63	1.10
				D6	0.41	0.72	0.57	1.10
		RES_010 10uM	NaCl 0.9%	D1	1.17	1.18	1.00	1.03
				D2	2.70	1.49	1.81	1.03
				D3	1.93	2.03	0.95	1.03
				D4	0.70	0.66	1.05	1.03
				D5	1.12	1.48	0.76	1.03
				D6	0.43	0.72	0.59	1.03
